# From Pyroptosis Heterogeneity to an Interpretable Prognostic Signature for Risk Stratification and Therapy Insights in Pancreatic Adenocarcinoma

**DOI:** 10.3390/biomedicines14040892

**Published:** 2026-04-14

**Authors:** Xiangsen Zou, Peng Song, Shicong Song, Guowei Zhang, Wang Xiao, Tingkang Yang, Lin Zhou, Yixiong Lin

**Affiliations:** 1 Department of Hepatobiliary Surgery, Nanfang Hospital, Southern Medical University, Guangzhou 510515, China; zxs14136@163.com (X.Z.); 13762043023@163.com (P.S.); wangxiao6248@163.com (W.X.); ytk18322807182@163.com (T.Y.); 2 Department of Geriatrics, Nanfang Hospital, Southern Medical University, Guangzhou 510515, China; songshc@smu.edu.cn; 3 Department of Hepatobiliary Surgery, Peking University Shenzhen Hospital, Shenzhen 518036, China; guoweizhang77@163.com; 4 Department of Endocrinology, Nanfang Hospital, Southern Medical University, Guangzhou 510515, China

**Keywords:** PAAD, pyroptosis heterogeneity, interpretable machine learning, prognostic model, tumor microenvironment, personalized therapy

## Abstract

**Background:** Pancreatic adenocarcinoma (PAAD) is a highly malignant cancer posing severe clinical challenges. Although the dual role of pyroptosis in tumor progression is increasingly recognized, the prognostic value of its molecular heterogeneity in PAAD remains underexplored. **Methods:** We integrated multi-omics data and applied interpretable machine learning to construct a predictive framework centered on pyroptosis heterogeneity. Using non-negative matrix factorization (NMF) on pyroptosis-related genes (PRGs), patients were classified into distinct molecular subtypes. Evaluating 117 machine learning combinations, we employed random survival forest (RSF) to build the final model, followed by comprehensive internal and external validation. SHapley Additive exPlanations (SHAP) analysis provided global and local interpretability. Clinical potential was assessed via nomogram, drug sensitivity prediction, single-cell analysis, and immunohistochemical validation. **Results:** We identified two biologically distinct pyroptosis subtypes and developed a ten-gene pyroptosis subtype-associated gene signature (PSAGS). PSAGS demonstrated robust performance across training, test, and multiple external validation cohorts, outperforming most published models. Multivariate analysis confirmed its independent prognostic value, and a PSAGS-based nomogram exhibited clinical utility. PSAGS-stratified subgroups showed differential responses to immunotherapy, chemotherapy, and targeted agents. Single-cell analysis revealed cell type-specific links between PSAGS scores and pyroptosis activity, indicating that high-PSAGS malignant cells foster an immunosuppressive microenvironment through extracellular matrix (ECM)-mediated signaling. Protein-level validation confirmed upregulation of signature genes in PAAD tissues. **Conclusions:** This work presents a biologically reliable prognostic model for personalized PAAD management and elucidates how pyroptosis heterogeneity drives tumor progression through cellular interactions.

## 1. Introduction

According to the Global Cancer Statistics 2022, pancreatic cancer poses a substantial global health burden, with an estimated 510,566 new cases and 467,005 deaths worldwide in 2022, ranking as the 12th most common cancer and the 6th leading cause of cancer death [1]. Pancreatic adenocarcinoma (PAAD), the most common histotype, is a highly lethal solid malignancy, exhibiting a mere 13% five-year overall survival (OS) rate; despite radical surgery, the postoperative five-year survival rate remains merely 17%, and the one-year recurrence rate is up to 48% [2]. Current clinical management faces significant challenges: chemotherapy has limited efficacy and frequent toxicities [3,4]; universal targeted therapies for key driver genes such as *KRAS* and *TP53* are lacking [5]; and immune checkpoint inhibitors show limited clinical benefit due to immunosuppressive microenvironment-mediated resistance mechanisms and low response rates [6]. These challenges underscore the urgent need to develop an accurate risk stratification system to guide clinical decisions and improve therapeutic outcomes.

A recent development is the study of pyroptosis, which serves as a critical mediator in bridging tumor cell demise and anti-tumor immune responses [7]. This process can be triggered by two major pathways: the canonical pathway involving inflammasome activation (e.g., *NLRP3*) and caspase-1-dependent cleavage of *GSDMD*; and the non-canonical pathway mediated by intracellular lipopolysaccharide and caspase-4/5/11, which also cleave *GSDMD* [8]. Through characteristic plasma membrane pore formation and inflammatory cytokine release, pyroptosis can both eliminate tumor cells and activate immunity, while its pro-inflammatory nature may also promote tumor growth [9]. The net impact of pyroptosis in PAAD is determined by the dynamic nature of its role as a pro-inflammatory versus an anti-cancer mechanism, but its molecular basis is highly inter-patient heterogeneous, which is a significant obstacle to clinical application [10]. In this study, we focus on analyzing the overall biological consequences of pyroptosis heterogeneity and translating them into a clinically actionable prognostic model, rather than distinguishing between specific pathways. Yet, most existing pyroptosis-based prognostic models directly use pyroptosis-related genes (PRGs) without first identifying molecular subtypes, thereby failing to capture the subtype-level heterogeneity of pyroptosis. Thus, the key to harnessing the anti-cancer potential of pyroptosis lies in developing more precise subtyping and predictive models that can cope with this heterogeneity.

This imperative has driven extensive research into the molecular subtyping of PAAD. However, such efforts remain exploratory, lacking a unified criterion for clinical decision-making [11]. Even though some studies have developed predictive models for PAAD, these are largely limited by the linear assumptions of classical methods such as Cox regression, which cannot reveal the complex non-linear interactions in high-dimensional data, thus restricting the predictive ability and generalizability of models [12]. Advances in explainable artificial intelligence have significantly enhanced our ability to analyze intricate biological data and reveal non-linear relationships among high-dimensional features, supporting the development of more robust tumor subtyping and predictive paradigms [13]. Here, we for the first time combine non-negative matrix factorization (NMF)-based molecular subtyping with interpretable machine learning to develop a prognostic model that captures pyroptosis heterogeneity.

We therefore leveraged multi-omics data to stratify PAAD patients into molecular subtypes by utilizing transcriptional signatures of PRGs and analyzed phenotypic disparities between subtypes. Using an explainable machine learning approach, we subsequently developed and verified a robust novel prognostic model, designated the pyroptosis subtype-associated gene signature (PSAGS), and evaluated its clinical applicability. Furthermore, by dissecting cellular interactions within the tumor microenvironment (TME) and the expression patterns of signature genes, we provided multi-layered evidence to demonstrate the biological plausibility of PSAGS. This research presents a validated prognostic framework to enable risk stratification and therapeutic decision-making in PAAD and elucidates how pyroptosis heterogeneity drives tumor progression through cellular interactions, thereby advancing its clinical translation toward personalized therapy.

## 2. Materials and Methods

### 2.1. Data Acquisition and Processing

Gene expression profiles for the TCGA-PAAD cohort were retrieved from the UCSC XENA database; clinical records and somatic mutation data for these cases were accessed via the TCGA GDC portal. Transcriptomic data from normal pancreatic tissues were sourced from the integrated TCGA-TARGET-GTEx dataset available in UCSC XENA [14]. Expression matrices and clinical metadata for cohorts GSE79668, GSE78229, GSE71729, GSE62452, and GSE183795 were obtained from the GEO database. Data for ICGC-PACA-AU and ICGC-PACA-CA cohorts were acquired through the SangerBox platform [15]. Expression data for the E-MTAB-6134 cohort were downloaded from the ArrayExpress repository. CPTAC-PAAD cohort data were retrieved from the cBioPortal database. Furthermore, we included 90 PRGs for investigation, obtained from the MSigDB database and prior literature [16,17,18,19]. The immune checkpoint gene set was referenced from published studies [17]. Detailed information for all cohorts and gene sets involved in this study is provided in Tables S1–S4.

The key steps in data processing included: (1) conversion and filtering of gene identifiers; (2) for GEO datasets, the “normalizeBetweenArrays” function from the “limma” R package was applied to standardize expression values across samples, reducing technical variations; (3) transformation of expression values using the log_2_(X + 1) formula; (4) exclusion of samples with an OS of <30 days. All cohorts were analyzed independently, and no additional batch correction was required.

### 2.2. Molecular Subtype Identification

Unsupervised clustering of the filtered PRG expression matrix was performed using NMF with the “NMF” R package. Selection of the optimal cluster count (k) was based on the cophenetic correlation coefficient, which was evaluated for k values between 2 and 10. The k value at which the coefficient exhibited the largest decline was selected, as this indicates a marked decline in cluster stability. To visualize inter-cluster separation, a two-dimensional scatter plot was generated using the results from principal component analysis (PCA) conducted with the “prcomp” function.

### 2.3. Molecular Features and Immune Microenvironment Analysis

These analyses aimed to characterize functional, genomic, and immune differences between the two pyroptosis-based molecular subtypes. Significant genes were identified via differential expression analysis using the “DESeq2” R package, with filtering criteria set at |log_2_FC| > 1 and FDR < 0.05, which are commonly used thresholds to balance sensitivity and specificity in transcriptomic studies. Functional enrichment analysis, covering both Gene Ontology (GO) terms and Kyoto Encyclopedia of Genes and Genomes (KEGG) pathways, was carried out using the “clusterProfiler” R package. A high-confidence protein–protein interaction (PPI) network was built using data from the STRING database (https://cn.string-db.org/, accessed on 8 April 2026) with a confidence score threshold of 0.7, including all available interactors without a size cutoff. No additional clustering analysis was performed. Subsequent visualization and topological analysis were performed in Cytoscape (v3.10.3). Hub genes were defined as those with a node degree of 15 or greater, based on the node degree distribution. Gene set variation analysis (GSVA) on Hallmark gene sets from the MSigDB database was performed using the “GSVA” R package to enable cross-subtype comparison of biological processes. Gene mutation profiles and tumor mutational burden (TMB) were assessed in samples with both transcriptome and somatic mutation data using the “maftools” R package. To analyze the tumor immune microenvironment, five algorithms (TIMER, MCPcounter, CIBERSORT, ESTIMATE, and ssGSEA) were utilized to comprehensively evaluate disparities in immune cell composition, stromal components, and immune-associated functional activity across groups.

### 2.4. Machine Learning Model Development

The “Mime” R package [20] was employed to develop prognostic models. Its development pipeline for PSAGS included: (1) Establishment of an initial feature set for model construction. These features are genes that are simultaneously associated with PAAD and prognosis, represent subtype differences, and contain core PRGs. (2) Generation of 117 prognostic models from different algorithm combinations under a leave-one-out cross-validation framework using the TCGA-PAAD cohort, followed by their evaluation on the testing cohorts (ICGC-PACA-AU, ICGC-PACA-CA, GSE79668, and GSE78229). (3) Among all testing cohorts, the highest mean C-index acted as the benchmark to identify the optimal prognostic model. Guided by this standard, the random survival forest (RSF) algorithm was selected. (4) Execution of two rounds of RSF modeling within the training cohort using the “randomForestSRC” R package. The first round aimed to screen key features based on variable importance and minimal depth. Minimal depth measures how early a gene is selected as a splitting variable in the decision trees. A smaller minimal depth indicates greater importance for survival prediction. The parameters were set as nsplit = 10, ntree = 1000, nodesize = 15, mtry = 5, and seed = 123,456. The second round rebuilt the model using the subset of features, with parameters adjusted to nsplit = 5, ntree = 1000, nodesize = 56, mtry = 3, and seed = 123,456.

### 2.5. Internal and External Model Validation

All internal and external validation analyses were performed on the final optimized 10-gene RSF model. To perform bootstrap optimism correction, we implemented a procedure involving 2500 iterations, each constructing a model with identical parameters. For each of the prognostic models, the optimism bias, computed using the “pec” R package to reflect the C-index discrepancy between the training cohort and out-of-bag validation cohorts, served to correct the initial C-index. The 95% confidence interval for the corrected C-index was then computed using the quantile method. To perform cross-validation, we used the “caret” R package to carry out 10-fold stratified sampling, which helped maintain consistent event proportions across folds. For each fold, we constructed a predictive model on the training subset and then calculated the C-index of this model on the corresponding test subset. This process yielded a summarized average C-index along with its 95% confidence interval, derived from the t-distribution. Subsequently, we sequentially calculated PSAGS scores for patients across the training cohort, four testing cohorts, and five independent external validation cohorts (GSE62452, GSE71729, GSE183795, E-MTAB-6134, and CPTAC-PAAD). Patients were stratified into high- and low-score groups based on the median PSAGS score, and we assessed the predictive accuracy at 1, 2, and 3 years. These time points were chosen because of the short median survival time of PAAD patients and the sharp decline in the number of patients with available follow-up data after 3 years, which would compromise the reliability of longer-term predictions. Furthermore, we systematically searched and collected 50 published PAAD prognostic models from PubMed (Table S5). Calculation of risk scores for each model across the ten cohorts employed the genes and coefficients from the original publications. This allowed for a direct, C-index-based comparison of the prognostic discriminatory power among the models.

### 2.6. Model Interpretation

The Shapley Additive exPlanations (SHAP) method determines the portion of each prediction attributable to individual features using game-theoretic Shapley values, providing both global and local explanations for the “black-box” mechanism of machine learning models [21]. This study employed the “kernelshap” R package to calculate SHAP values and the “shapviz” R package for visualization. By interpreting the direction and magnitude of SHAP values, we assessed the impact of individual genes on model predictions, and visualized gene importance rankings, the relationship between gene expression levels and predictions, and individual contribution patterns.

### 2.7. Nomogram Development and Evaluation

A nomogram incorporating independent prognostic factors for predicting 1-, 2-, and 3-year OS was constructed using the “rms” R package. Its performance was evaluated through internal validation, calibration curve analysis, time-dependent receiver operating characteristic (ROC) assessment, and decision curve analysis (DCA).

### 2.8. Therapeutic Response Prediction

Per-sample potential responses to immune checkpoint blockade were assessed using the Tumor Immune Dysfunction and Exclusion (TIDE) algorithm. The generated TIDE scores were then compared across PSAGS-defined subgroups to infer relative immune escape probabilities. Drug sensitivity was evaluated via the “oncoPredict” R package, which leverages a pre-trained model from the GDSC2 database (https://www.cancerrxgene.org/, accessed on 8 April 2026) to calculate the half-maximal inhibitory concentration (IC50) values for a broad range of therapeutic agents based on tumor transcriptome profiles. Through inter-group comparison, a drug was identified as a candidate therapeutic for a specific PSAGS score group if it exhibited a significantly lower median predicted IC50 value within that group.

### 2.9. Single-Cell Data Quality Control and Annotation

Single-cell RNA sequencing (scRNA-seq) data from pancreatic ductal adenocarcinoma (PDAC) samples in the GSE212966 dataset [22] were processed using the “Seurat” R package (v5.3.0). Quality control criteria included: expression in ≥3 cells per gene; ≥200 detected genes per cell; nCount_RNA between 1000 and 40,000; nFeature_RNA between 500 and 6000; mitochondrial gene percentage < 22%; and hemoglobin gene percentage < 1%. Following removal of doublets and ambient RNA contamination, cell cycle effects were regressed out during normalization and scaling. Batch effects were corrected using Harmony. After PCA dimensionality reduction, cells were clustered and visualized using uniform manifold approximation and projection (UMAP) or t-distributed stochastic neighbor embedding (t-SNE). Cluster-specific differentially expressed genes (DEGs) were identified, and cell types were manually annotated based on canonical markers from the literature [22,23].

### 2.10. Malignant Ductal Cell and Subgroup Identification

Using the “copykat” R package (v1.1.0) [24] with its default parameters and intra-sample T, B, and neutrophil cells as diploid references, malignant ductal cells were determined through copy number variation (CNV) analysis. Cells identified as “aneuploid” were defined as malignant. A dedicated analysis workflow was then implemented for these malignant cells aimed at delineating intrinsic subgroups, including re-normalization, selection of highly variable genes, batch correction, PCA, and clustering. To characterize these subgroups, marker genes for each were identified and used for GO biological process (BP) enrichment analysis.

### 2.11. Pyroptosis Activity and Cellular PSAGS Score Quantification

Pyroptosis activity was quantified by calculating enrichment scores for the set of PRGs, utilizing the “AddModuleScore_UCell” function provided by the “UCell” R package (v2.10.1) [25]. The PSAGS score per cell was computed by applying the established prognostic model using the “predict” function. For subsequent analyses, all cells or specified cell populations were divided into groups with high scores and those with low scores, with the median PSAGS score serving as the threshold.

### 2.12. Cell Communication Analysis

The “CellChat” R package (v1.6.1) was employed to analyze cell–cell communication [26]. Based on the normalized expression matrix and the CellChatDB.human database, communication probabilities at the cellular and pathway levels were calculated and compared between groups. To identify significant differential ligand–receptor pairs between malignant ductal cells of the groups with high versus low PSAGS scores and microenvironmental cells, the following criteria were applied: First, a pair was considered differential if it satisfied either criterion: (1) a fold change (FC) > 1.5 for communication from groups with high scores versus those with low scores, with statistically significant cell–cell crosstalk detected in the former; or (2) FC < 0.67, with notable intercellular signaling observed in the latter. Second, the maximum communication probability of the pair across the two groups was required to exceed the 75th percentile of all compared pairs. Finally, all pairs meeting these conditions were ranked in descending order by |log_2_FC|, and the top 50 were selected for visualization.

### 2.13. Immunohistochemistry

Evaluation of GSDMC protein expression employed four paired PAAD tumor and adjacent normal tissue specimens obtained from Nanfang Hospital, Southern Medical University. After dewaxing, 4 μm sections underwent citrate-based antigen retrieval and subsequent blocking against endogenous peroxidase and nonspecific binding. Tissue sections were incubated overnight at 4 °C with the primary antibody against GSDMC (Proteintech, 30469-1-AP, 1:500). Detection was performed by sequential application of a biotinylated secondary antibody and streptavidin–biotin complex, DAB development, hematoxylin counterstaining, and neutral gum mounting. GSDMC immunohistochemical staining was quantified in ImageJ (v1.54f) by measuring the integrated optical density per unit area (IOD/Area). Additionally, the Human Protein Atlas (HPA; https://www.proteinatlas.org/, accessed on 8 April 2026) was used to investigate protein expression patterns of other relevant genes.

### 2.14. Statistical Analysis

R software (v4.4.2) was employed for statistical analyses and data visualization. Continuous variables were compared between two groups via the Wilcoxon rank-sum test, while the Kruskal–Wallis test was applied for multi-group comparisons. For categorical variables, either the chi-square test or Fisher’s exact test was utilized depending on expected counts. Bivariate associations were evaluated using Spearman’s rank correlation coefficient. Statistical significance was defined as *p* < 0.05, with figure annotations: ns (*p* > 0.05), * (*p* < 0.05), ** (*p* < 0.01), *** (*p* < 0.001).

### 2.15. The Workflow Chart of This Study


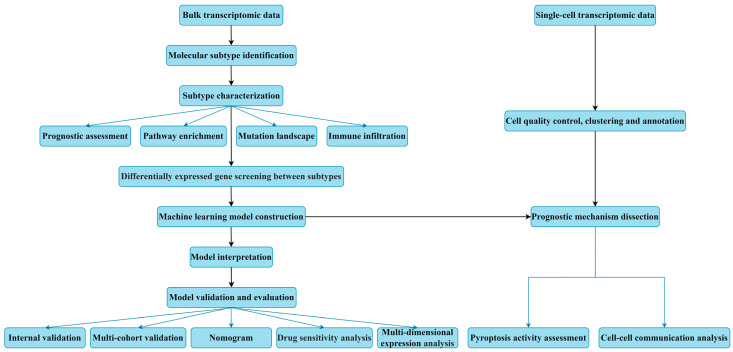


## 3. Results

### 3.1. Identification of Pyroptosis Molecular Subtypes in PAAD

**Figure 1:**
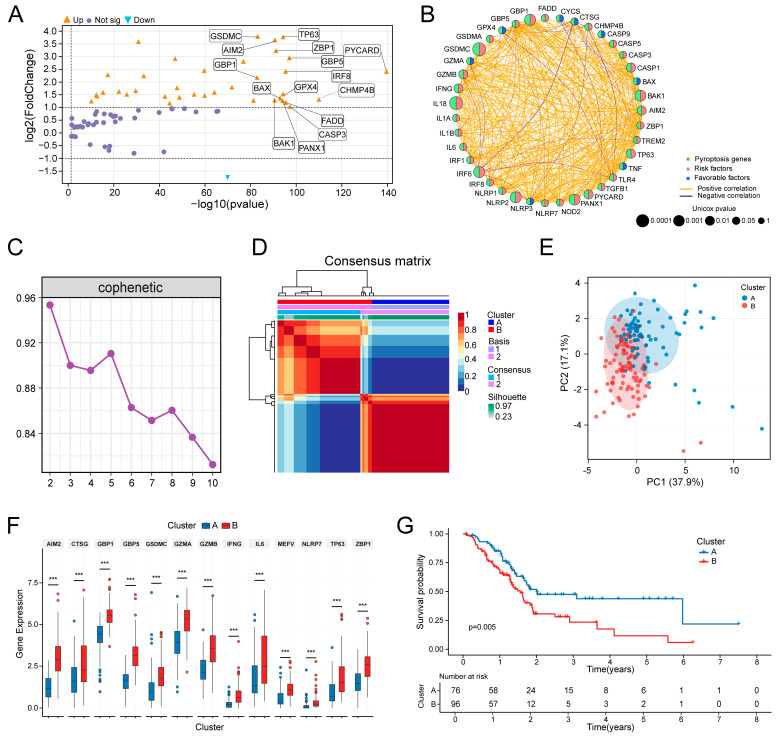
Identification of pyroptosis-related molecular subtypes in pancreatic cancer via non-negative matrix factorization (NMF). (**A**) Volcano plot of differentially expressed pyroptosis-related genes (DEPRGs) between tumor and normal tissues, with the labeled genes used for subtyping. (**B**) Prognostic network of DEPRGs, wherein genes with *p* < 0.001 were utilized for subtyping. (**C**) Cophenetic coefficient curve for the optimal cluster count (k) ranging from 2 to 10. (**D**) Consensus matrix of the NMF clustering at k = 2. (**E**) Principal component analysis (PCA) plot between clusters. (**F**) Box plots of DEPRGs between clusters. (**G**) Kaplan–Meier (KM) survival curves between clusters. Statistical methods: Wilcoxon rank-sum test (**F**); log-rank test (**G**); *** (*p* < 0.001).

Transcriptomic profiling of PAAD versus normal tissues identified 7193 DEGs (Figure S1A). Intersection with 90 PRGs yielded 39 differentially expressed PRGs (DEPRGs) (Figure S1B), of which 38 were upregulated, and only 1 was downregulated in tumors (Figure 1A). Functional enrichment of DEPRGs was observed in the inflammatory response, programmed cell death, and signaling pathways, including NOD-like receptor, NF-κB, and TNF (Figure S1C). Hub genes, including *CASP1*, *IL18*, and *TNF*, were identified via PPI network analysis (Figure S1D). Univariate Cox regression screening yielded 13 genes with significant prognostic relevance (Figure 1B). For these genes, Kaplan–Meier (KM) survival analysis showed that upregulation of 12 (excluding *PYCARD*) correlated significantly with poorer patient outcomes (*p* < 0.05, Figure S2).

To establish a pyroptosis-based molecular classification for PAAD, we first defined a 16-key-PRG set. This set was derived by integrating the two prognostic genes from Cox regression (*p* < 0.001) with the 15 most significantly differentially expressed DEPRGs (Table S6). Using these genes, we performed NMF clustering on 178 PAAD patients from the TCGA cohort. Based on the cophenetic coefficient, the optimal cluster number was determined to be two (Figure 1C,D). Accordingly, patients were stratified into Cluster A and Cluster B, with *n* = 80 and *n* = 98, respectively. The clear separation trend between the two subtypes was confirmed by PCA (Figure 1E). Differential analysis further identified 13 specific PRGs that were significantly upregulated in Cluster B (Figure 1F), suggesting enhanced pyroptotic activity in this cluster. In terms of clinicopathological features, comparison revealed a significant difference only in tumor grade, with Cluster B having a higher proportion of high-grade (G2/G3) tumors (Figure S3). Finally, KM analysis demonstrated that assignment to Cluster A was associated with a significant OS advantage over Cluster B (*p* = 0.005, Figure 1G), highlighting the clear prognostic discriminative power of this classification.

### 3.2. Differential Analysis of Molecular Features and Immune Landscapes Between Subtypes

**Figure 2:**
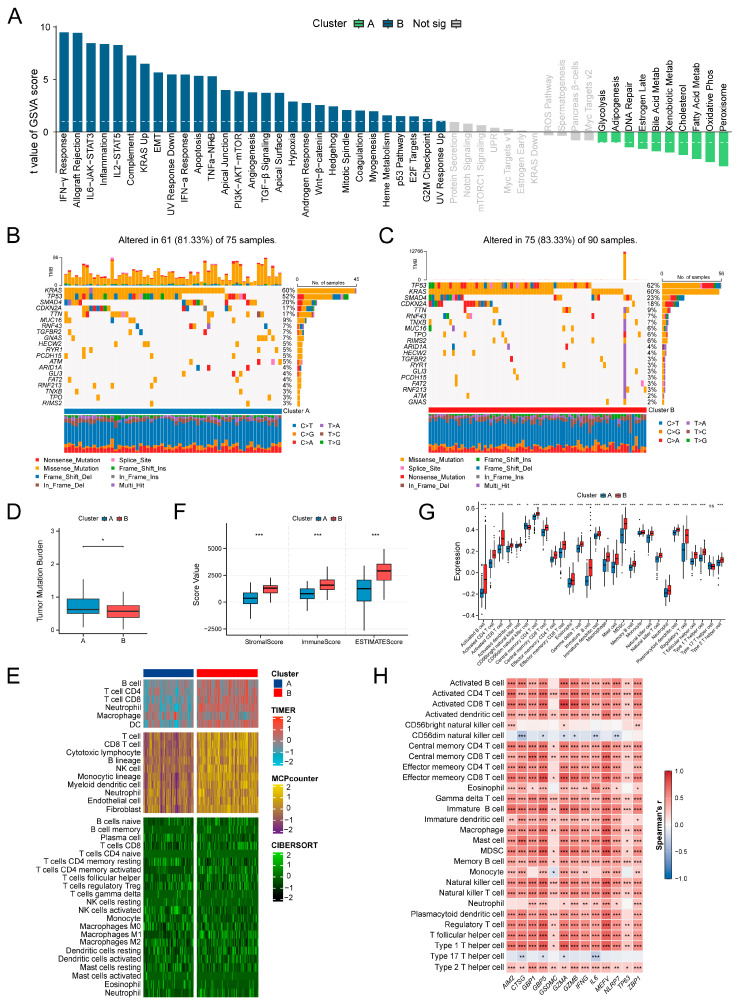
Molecular features and immune landscape of pyroptosis-related molecular subtypes. (**A**) Gene set variation analysis (GSVA) enrichment bar plot between Cluster A and Cluster B. (**B**) Waterfall plot of gene mutations in Cluster A. (**C**) Waterfall plot of gene mutations in Cluster B. (**D**) Comparison of tumor mutational burden (TMB) between clusters. (**E**) Immune cell infiltration levels between clusters evaluated by multiple algorithms. (**F**) Comparison of ESTIMATE scores between clusters. (**G**) Differences in immune cell enrichment scores between clusters based on single-sample gene set enrichment analysis (ssGSEA). (**H**) Spearman correlation heatmap between DEPRGs in clusters and immune cells. Statistical methods: Wilcoxon rank-sum test (**D**,**F**,**G**); Spearman correlation (H); ns (*p* > 0.05), * (*p* < 0.05), ** (*p* < 0.01), *** (*p* < 0.001).

To characterize functional, genomic, and tumor immune microenvironmental differences between the two pyroptosis-based subtypes, we began by performing GSVA. Primary enrichments in immune-related and pro-tumorigenic pathways, including IL6-JAK-STAT3 signaling, inflammatory response, epithelial–mesenchymal transition (EMT), KRAS signaling, and apoptosis, were observed in Cluster B. In contrast, Cluster A was characterized by the predominant enrichment of metabolic pathways such as glycolysis, fatty acid metabolism, and oxidative phosphorylation (Figure 2A). Subsequent mutation analysis revealed that common genes, including *KRAS*, *TP53*, *SMAD4*, *TTN*, and *CDKN2A*, were frequently mutated in both subtypes, with missense mutations and C>T substitutions being predominant. However, the highest-frequency mutations were subtype-specific: *KRAS* in Cluster A and *TP53* in Cluster B (Figure 2B,C). Notably, Cluster A exhibited a significantly higher TMB than Cluster B (Figure 2D), suggesting greater genomic instability potentially associated with its metabolically active microenvironment.

Compared to Cluster A, Cluster B exhibited enhanced infiltration of diverse immune cell types, such as B cells, T cells, neutrophils, and dendritic cells (Figure 2E), and concurrently displayed markedly elevated immune and stromal scores (Figure 2F). To validate this, single-sample gene set enrichment analysis (ssGSEA) was performed, revealing higher enrichment levels for most immune cell types in Cluster B, with the exception of Type 17 T helper cells and CD56dim natural killer cells (Figure 2G). Spearman correlation analysis identified a significant positive correlation between these abundant immune cells and most of the upregulated PRGs in Cluster B (Figure 2H). These results outline an immune-activated phenotype for Cluster B and imply a potential role for PRGs in modulating both immune infiltration and stromal activation, which may underlie the observed prognostic difference between the subtypes.

### 3.3. Construction and Validation of a Pyroptosis Subtype-Associated Prognostic Model via Interpretable Machine Learning

To develop a prognostic model capitalizing on inter-subtype differences, we first identified 1181 differentially expressed pyroptosis subtype-associated genes (DEPSGs) between Clusters A and B (Figure S4A). Subsequently, we screened for prognostically relevant genes within the training cohort via univariate Cox regression analysis (*p* < 0.05) and validated the consistency of their prognostic trends across all test cohorts with a lenient threshold (*p* < 0.5), yielding 367 stable prognosis-related genes. Intersecting these 367 genes with both the 1181 DEPSGs and tumor-versus-normal DEGs refined the list to 25 core genes (Figure S4B). In parallel, the intersection of the 13 previously identified prognosis-related DEPRGs with the 13 PRGs significantly upregulated in Cluster B yielded 6 core PRGs (Figure S4C). Finally, combining the 25 core genes and the 6 core PRGs produced a candidate set of 29 DEPSGs (Figure S4D).

**Figure 3:**
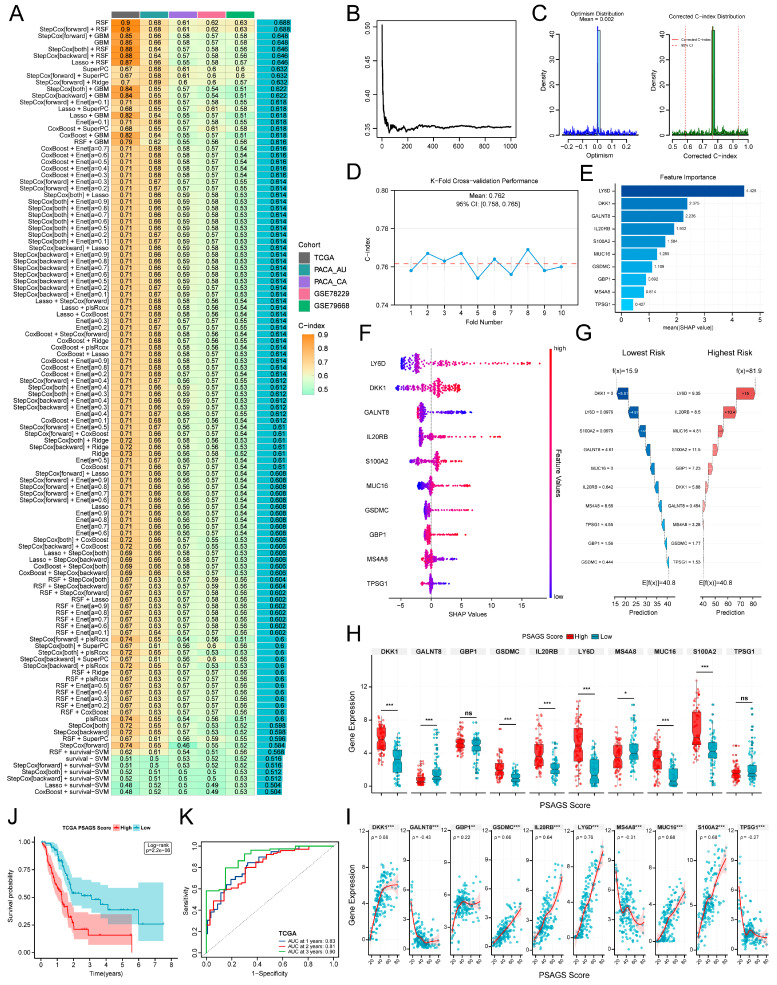
Development and validation of pyroptosis subtype-associated gene signature (PSAGS) using explainable machine learning. (**A**) C-index of different algorithm combinations in training and test cohorts. (**B**) Error rate curve of the final random survival forest (RSF) model. (**C**) Distribution of the optimism and corrected C-index for the model based on bootstrap validation in the training cohort. (**D**) Distribution of the C-index for the model based on 10-fold cross-validation in the training cohort. (**E**) Bar plot of global feature importance based on Shapley Additive exPlanations (SHAP) values. (**F**) Beeswarm plot of SHAP values distribution for each feature. (**G**) SHAP waterfall plots explaining predictions for the highest-risk and lowest-risk patients. (**H**) Comparison of model gene expression levels between high and low PSAGS score groups. (**I**) Correlation analysis between model gene expression and PSAGS scores. (**J**) KM survival plots for PSAGS score-stratified groups. (**K**) Time-dependent ROC curves estimating 1–3 years overall survival (OS). Statistical methods: Wilcoxon rank-sum test (**H**); Spearman correlation (**I**); log-rank test (**J**); ns (*p* > 0.05), * (*p* < 0.05), ** (*p* < 0.01), *** (*p* < 0.001).

Evaluation of 117 algorithm combinations in the training cohort, based on the candidate gene set, revealed RSF as the optimal model, which showed stable and high performance across all test cohorts (mean C-index: all cohorts, 0.688; training cohort, 0.9; test cohorts, 0.635) (Figure 3A). Consequently, using RSF, we constructed an initial model based on the DEPSGs and applied a minimal depth threshold (<6.9254) to screen ten key genes: *LY6D*, *DKK1*, *GALNT8*, *IL20RB*, *S100A2*, *MUC16*, *GSDMC*, *GBP1*, *MS4A8*, and *TPSG1* (Figure S4E). These ten genes represent the core DEPSGs and collectively reflect the molecular heterogeneity of pyroptosis. Among them, *GSDMC* and *GBP1* are additionally implicated in pyroptosis regulation. A refined RSF model rebuilt and optimized using these ten genes achieved a stable error rate of approximately 35% (Figure 3B). To evaluate the robust performance of the prognostic model, we performed stringent validation via two complementary approaches. First, following 2500 bootstrap resamples, the model produced a calibrated C-index of 0.767 (95% CI: 0.585–0.937) with a mean optimism of 0.002 (Figure 3C). Second, through 10-fold cross-validation, it generated a C-index of 0.762 (95% CI: 0.758–0.765) (Figure 3D). These results verify that the model possesses strong predictive and generalization ability. Based on this, we selected this optimized 10-gene RSF model as the final version, designating it the PSAGS.

Next, to interpret the PSAGS and identify key prognostic drivers, we applied SHAP analysis. LY6D showed the greatest contribution to risk prediction, with a mean absolute SHAP value of 4.428. By comparison, the PRGs *GSDMC* and *GBP1* had mean absolute SHAP values of 1.109 and 0.892, respectively (Figure 3E). For most genes (except *GALNT8*, *MS4A8*, and *TPSG1*), higher expression correlated with higher SHAP values (Figure 3F), suggesting an association with increased mortality risk. We further verified this trend in subsequent differential expression profiling and correlation assessments between groups stratified by PSAGS score dichotomization (Figure 3H,I). To illustrate how the model operates at the level of individual patients, we visualized the SHAP values for each gene in representative patients (Figure 3G). Positive SHAP values increase the predicted risk, while negative values decrease it. This allows clinicians to identify which genes drive high-risk classification in a patient, potentially guiding personalized follow-up or treatment decisions. We then confirmed the prognostic validity of the PSAGS. KM analysis revealed a significant survival disadvantage for patients with high PSAGS scores relative to those with low scores (*p* < 0.001, Figure 3J). The model also demonstrated excellent time-dependent discriminative ability in the training cohort, with AUCs of 0.83 at 1 year, 0.81 at 2 years, and 0.90 at 3 years for prognostic prediction (Figure 3K). Notably, the PSAGS displayed a strong biological linkage to our initial molecular subtypes. Specifically, 66.3% of patients with high PSAGS scores belonged to Cluster B (Figure S4F), and PSAGS scores were markedly elevated in Cluster B compared to Cluster A (Figure S4G). Collectively, these findings position PSAGS as a potential robust prognostic tool while providing additional evidence for the more aggressive biological nature of Cluster B.

### 3.4. Validation of PSAGS Across Multiple Cohorts

**Figure 4:**
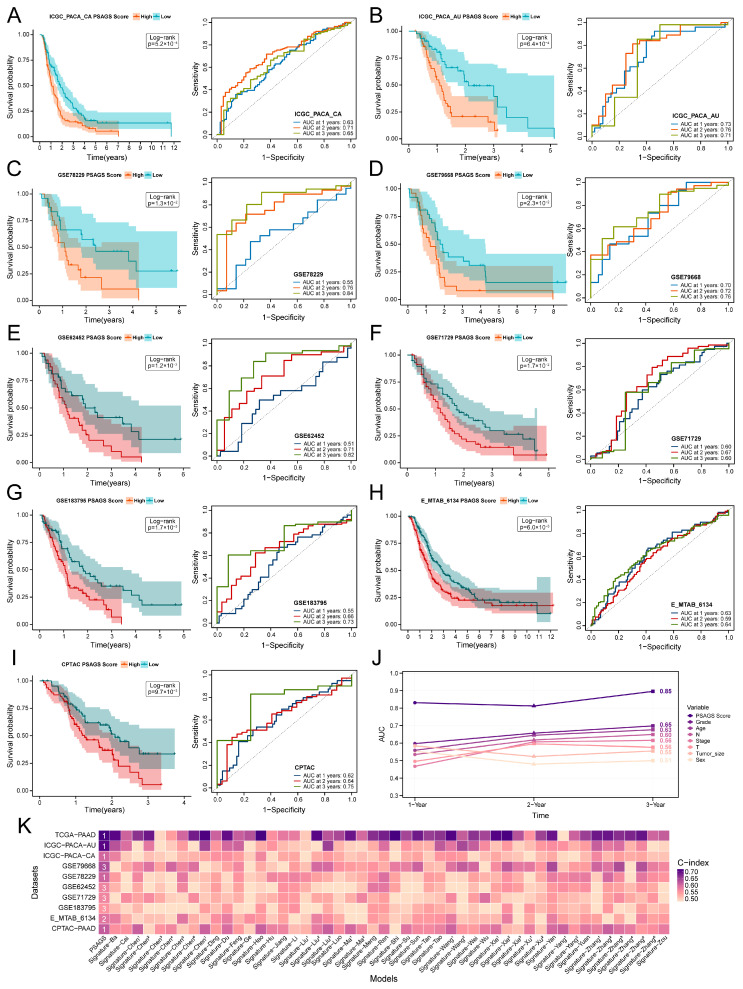
Multicohort validation of the prognostic performance of PSAGS. (**A**–**D**) Performance validation of PSAGS in four test cohorts. (**E**–**I**) External validation of PSAGS in five independent cohorts. (**J**) 1–3 years OS prediction area under the curve (AUC) comparison: PSAGS and traditional clinical indicators in training cohort. (**K**) Heatmap of C-index comparison: PSAGS and 50 published models, with numbers showing ranking. Statistical methods: log-rank test (**A**–**I**).

We performed a systematic assessment of the predictive performance of PSAGS. First, we divided patients into a high-score group and a low-score group using the median PSAGS score as the threshold. Initial analysis indicated a trend of shortening patient survival time with increasing PSAGS score (Figure S5). This trend was definitively confirmed by KM analysis, which revealed significantly shorter OS in the high-score group across four test cohorts (log-rank *p* < 0.05, Figure 4A–D). Consistent survival differences were also observed in five external validation cohorts (Figure 4E–I), which include datasets from different platforms and sample sizes ranging from 64 to 309 patients, demonstrating the model’s robustness across heterogeneous datasets. Subsequently, we assessed the prognostic accuracy of the model using ROC curve analysis, which exhibited robust prognostic utility over 1-, 2-, and 3-year follow-up windows. Specifically, the test cohorts yielded AUC values of: ICGC-PACA-CA (0.63, 0.71, 0.65); ICGC-PACA-AU (0.73, 0.76, 0.71); GSE78229 (0.55, 0.76, 0.84); and GSE79668 (0.70, 0.72, 0.76) (Figure 4A–D). Among external validation cohorts, AUC values were: GSE62452 (0.51, 0.71, 0.82); GSE71729 (0.60, 0.67, 0.60); GSE183795 (0.55, 0.66, 0.73); E-MTAB-6134 (0.63, 0.59, 0.64); and CPTAC-PAAD (0.62, 0.64, 0.75) (Figure 4E–I).

In addition, the mean 3-year prediction AUC of PSAGS in the training cohort reached 0.85, surpassing traditional clinical predictors including age, sex, tumor stage, and histologic grade (Figure 4J). A comparative analysis with 50 published PAAD prognostic models revealed that the C-index of PSAGS ranked among the top three across all evaluated cohorts (Figure 4K). These findings demonstrate that PSAGS is an excellent tool for prognostic assessment in PAAD.

### 3.5. Analysis of Clinical Correlates and Construction of a Prognostic Nomogram for PSAGS

**Figure 5:**
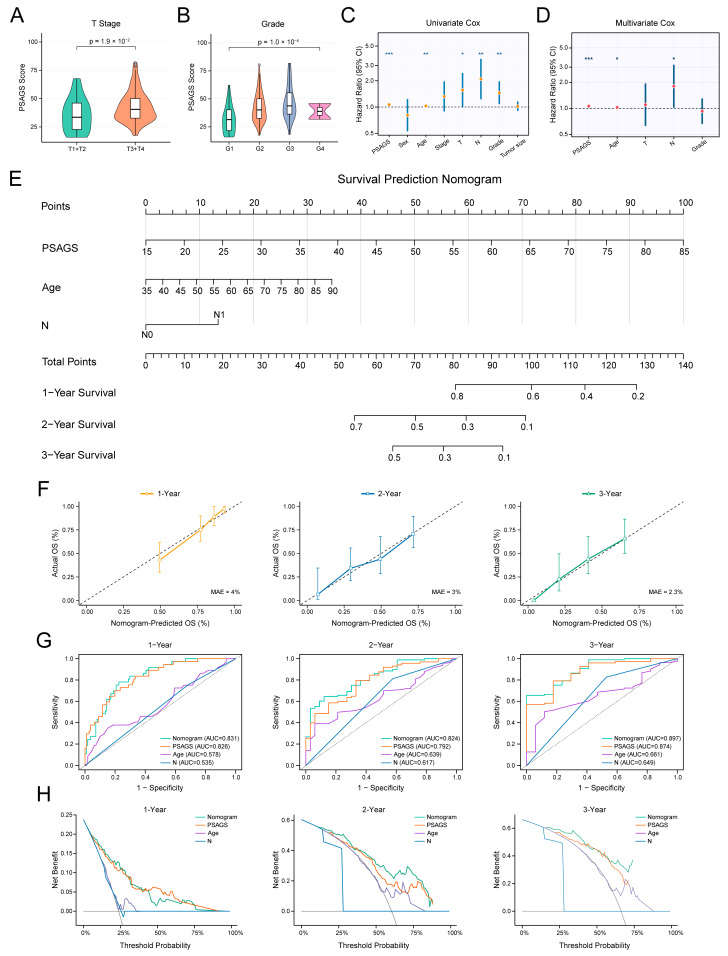
Evaluation of PSAGS clinical correlations and the prognostic nomogram. (**A**) Distribution of PSAGS scores across different T stages. (**B**) Distribution of PSAGS scores across different histological grades. (**C**) Univariate Cox regression for OS. (**D**) Multivariate Cox regression for OS. (**E**) Nomogram for predicting 1–3 years OS based on independent prognostic factors. (**F**) Calibration curves of the nomogram for evaluating 1–3 years OS. (**G**) Time-dependent receiver operating characteristic (ROC) curves for evaluating 1–3 years OS. (**H**) Decision curves for evaluating 1–3 years OS. Statistical methods: Wilcoxon rank-sum test (**A**); Kruskal–Wallis test (**B**); Wald test from Cox regression (**C**,**D**); * (*p* < 0.05), ** (*p* < 0.01), *** (*p* < 0.001).

We proceeded to evaluate the clinical utility of PSAGS by examining its relationship with clinicopathological features and constructing a corresponding nomogram. Stratified analysis indicated considerable differences in PSAGS scores with respect to T stage and histologic grade, with scores increasing as the disease progressed (Figure 5A,B). This pattern suggests a potential relationship between PSAGS score and tumor aggressiveness. In contrast, PSAGS scores did not differ significantly across subgroups defined by age, sex, stage, N stage, or tumor size (Figure S6A). PSAGS score was identified as a candidate prognostic factor in univariate regression, and further confirmed as an independent predictor (HR = 1.06, 95% CI: 1.04–1.08, *p* < 0.001) alongside age and N stage in multivariate regression (Figure 5C,D). We then constructed a nomogram based on these three factors (Figure 5E). For example, a patient with a PSAGS score of 50 (50 points), age of 67 years (20 points), and N0 stage (0 points) would have a total of 70 points, corresponding to 1-, 2-, and 3-year survival probabilities of approximately 90%, 50%, and 42%, respectively. Internal validation of the nomogram with 2500 bootstrap resamples yielded a calibrated C-index of 0.734 (95% CI: 0.623–0.838) with an optimism of 0.013 (Figure S6B). Nomogram calibration curve analysis showed strong concordance between predicted and observed OS. The corresponding estimation errors for 1-, 2-, and 3-year predictions were 4%, 3%, and 2.3% (Figure 5F). In ROC analysis, the nomogram achieved higher AUC values for 1- to 3-year predictions compared to the PSAGS score alone, and it also significantly surpassed the performance of age and N stage (Figure 5G). Throughout a broad spectrum of threshold probabilities in DCA, the nomogram yielded a clinical net benefit comparable to the PSAGS score and greater than that of other clinical variables, particularly for longer-term predictions (Figure 5H). Specifically, for 2- and 3-year predictions, the nomogram provides a higher net benefit than the PSAGS score alone for threshold probabilities approximately between 0.1 and 0.8, while for 1-year predictions the advantage is modest and limited to low thresholds (0.1–0.4). Collectively, these results support the utility of the nomogram in guiding clinical decisions.

### 3.6. Predictive Value of PSAGS for Immunotherapy Response and Drug Sensitivity

**Figure 6:**
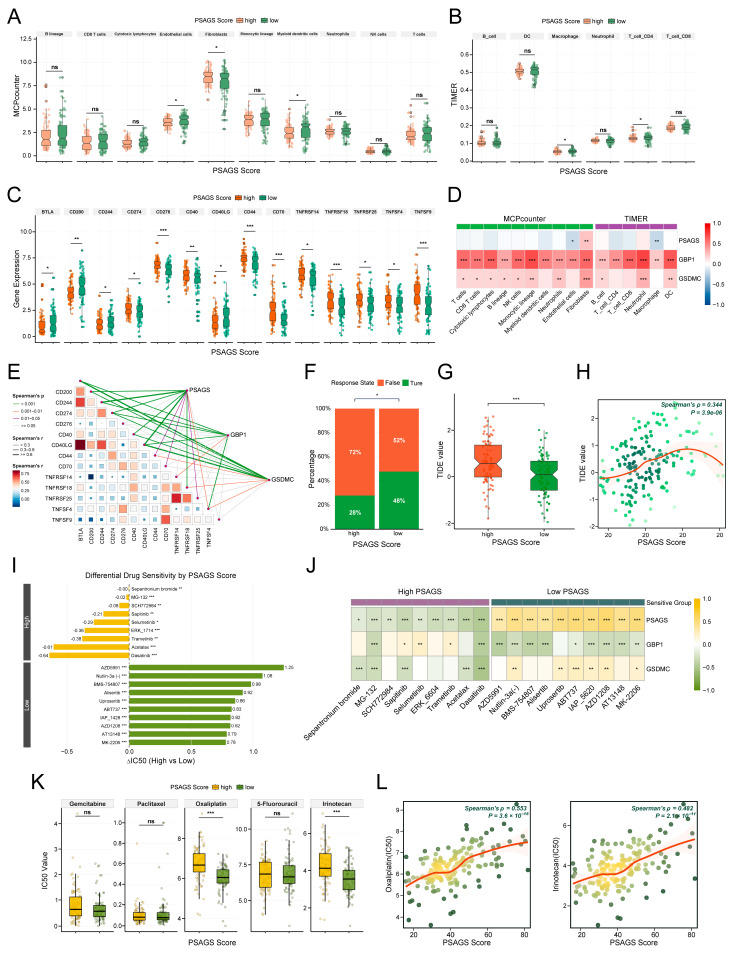
Assessment of PSAGS in predicting immunotherapy response and drug sensitivity. (**A**) MCPcounter analysis of immune cell infiltration in high and low PSAGS score groups. (**B**) TIMER-based group comparison of immune cell infiltration. (**C**) Group comparison of immune checkpoint molecule expression. (**D**) Heatmap of the correlations among PSAGS score, core pyroptosis genes *GSDMC* and *GBP1*, and immune cell infiltration. (**E**) Heatmap of the correlations among PSAGS score, core genes, and immune checkpoint molecule expression. (**F**) Group comparison of immunotherapy response rates. (**G**) Group comparison of Tumor Immune Dysfunction and Exclusion (TIDE) values. (**H**) Scatter plot showing correlation between PSAGS score and TIDE value. (**I**) Top 10 sensitive drugs for high and low PSAGS groups based on differences in median half-maximal inhibitory concentration (IC50) values. (**J**) Heatmap of the correlations among PSAGS score, core genes, and IC50 values of sensitive drugs. (**K**) Group comparison of IC50 values for common chemotherapeutic drugs. (**L**) Scatter plot for correlation analysis of PSAGS score with oxaliplatin/irinotecan IC50 values. Statistical methods: Wilcoxon rank-sum test (**A**–**C**,**G**,**I**,**K**); Spearman correlation (**D**,**E**,**H**,**J**,**L**); chi-square test (**F**); ns (*p* > 0.05), * (*p* < 0.05), ** (*p* < 0.01), *** (*p* < 0.001).

We evaluated the translational potential of PSAGS by investigating its links to immunotherapy response and agent sensitivity. Inter-group comparison revealed distinct immunophenotypes: patients with low PSAGS scores had an enriched milieu of CD4+ T cells, endothelial cells, myeloid dendritic cells, and macrophages, while those with high scores had a landscape dominated by fibroblasts (Figure 6A,B). This pattern suggests a more active immune microenvironment in the former, contrasting with a potentially immunosuppressive state in the latter. Furthermore, the high score group had substantially higher expression of the immune checkpoints *CD274* and *CD276*, as well as tumor progression factors *TNFRSF14* and *TNFSF4*. Conversely, the low-score group exhibited higher expression levels of immune modulators *BTLA* and *CD200*, as well as co-stimulatory molecules *CD244* and *CD40LG* (Figure 6C). While the PSAGS score itself was not significantly associated with most immune cell infiltration levels, the PRGs *GSDMC* and *GBP1* within the model correlated positively with the infiltration of multiple cell types, including neutrophils, dendritic cells, CD8+ T cells, and fibroblasts (Figure 6D). PSAGS score and both genes also correlated positively with the expression of most immune checkpoint molecules (Figure 6E), indicating that the PSAGS-defined immunosuppressive phenotype may involve coordinated upregulation of these checkpoints by *GSDMC* and *GBP1*. More importantly, the immunotherapy response rate was significantly reduced in patients carrying high PSAGS scores (28%) relative to those having lower scores (48%) (Figure 6F). A concomitant increase in TIDE scores, positively correlated with PSAGS scores, was also noted in the high-score group (Figure 6G,H). These data point to a patient population with lower PSAGS scores as potentially more responsive to immunotherapy.

In drug sensitivity analysis, the two groups differed significantly in IC50 for 142 out of 198 anti-tumor drugs. Specifically, among the top-sensitive agents for each group (Figure 6I), dasatinib and trametinib were more potent in patients with high PSAGS scores, in contrast to AZD5991 and Nutlin-3a, which demonstrated greater efficacy in those with low scores. An inverse correlation between PSAGS scores and the IC50 of drugs to which the high score group was sensitive was observed, whereas a positive correlation was found for agents effective in the low score group. *GSDMC* exhibited sensitivity patterns largely aligned with PSAGS, whereas *GBP1* demonstrated a predominantly opposite trend (Figure 6J). Notably, among commonly used chemotherapeutic agents for PAAD, the low PSAGS score group exhibited higher sensitivity to oxaliplatin and irinotecan, and this sensitivity was further enhanced with decreasing scores. For gemcitabine, 5-fluorouracil, and paclitaxel, no significant differences in sensitivity between groups were detected (Figure 6K,L). Overall, these results support PSAGS as a potential reference to guide individualized drug therapy in PAAD patients.

### 3.7. Single-Cell Profiling of Cell Type-Specific Inverse Regulation Between PSAGS Score and Pyroptotic Activity in PDAC

**Figure 7:**
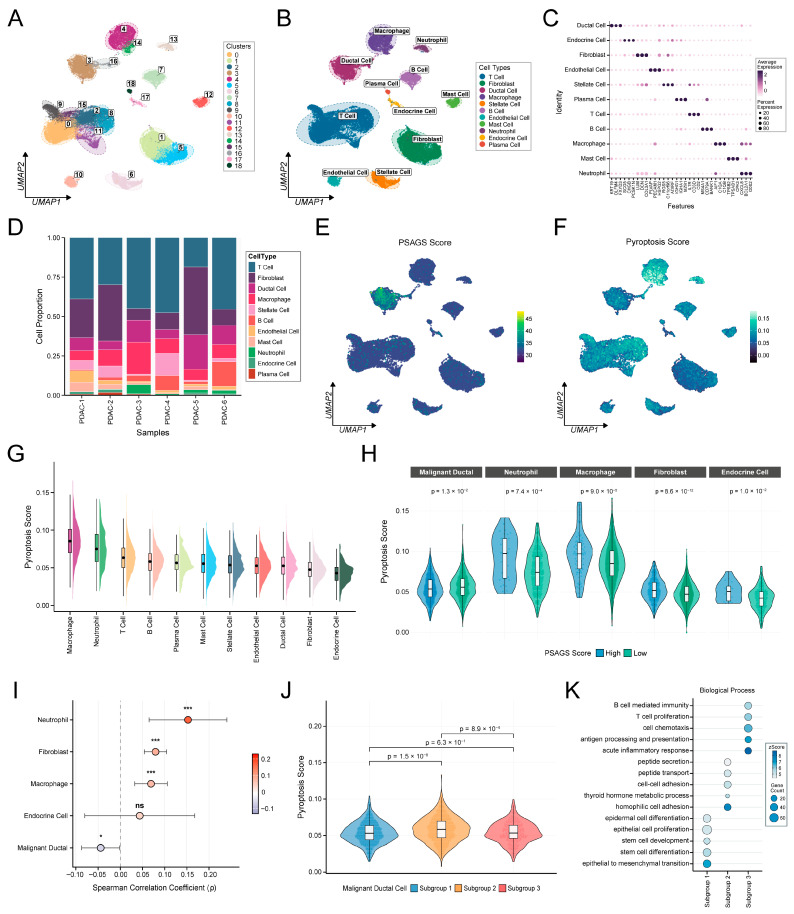
Single-cell dissection of cell type-specific relationships between PSAGS score and pyroptosis activity in pancreatic ductal adenocarcinoma (PDAC). (**A**) Uniform manifold approximation and projection (UMAP) plot showing cell clusters. (**B**) UMAP plot for cell type annotations. (**C**) Bubble plot of cell type marker genes. (**D**) Cellular composition across samples. (**E**) Distribution of PSAGS scores in UMAP space. (**F**) Distribution of PRGs enrichment scores in UMAP space. (**G**) Raincloud plots of PRGs enrichment scores across cell types. (**H**) Cell types exhibiting significant differences in PRGs enrichment scores between high and low PSAGS groups within each type. (**I**) Correlation between PSAGS scores and PRGs enrichment scores in specific cell types. (**J**) Comparison of PRGs enrichment scores among malignant ductal cell subgroups. (**K**) Enrichment analysis of biological processes in malignant ductal cell subgroups. Statistical methods: Wilcoxon rank-sum test (**H**,**J**); Spearman correlation (**I**); ns (*p* > 0.05), * (*p* < 0.05), *** (*p* < 0.001).

We explored the relationship between PSAGS score and pyroptotic activity using scRNA-seq data from six PDAC samples. Following quality control (Figure S7A), we obtained 27,656 high-quality cells. Based on the elbow plot result (Figure S7B), clustering with the top 20 principal components identified 19 cell clusters (Figure 7A). Using known marker genes, these clusters were assigned to 11 cell types, including ductal cells, fibroblasts, T cells, and macrophages (Figure 7B). The top three marker genes for each type are shown in Figure 7C. Although cellular composition ratios varied among samples, all were dominated by these four cell types (Figure 7D). Through CNV analysis, we identified 2077 malignant ductal cells and further subdivided them into three subgroups (Figure S7C,D).

Across cell populations, ductal cells showed significantly higher PSAGS scores than other non-malignant cells (Figure 7E). Macrophages exhibited the highest pyroptotic activity, followed by neutrophils, T cells, and B cells (Figure 7F,G). Notably, the relationship between PSAGS score and pyroptotic activity varied by cell type. In malignant ductal cells, the high PSAGS score group displayed lower pyroptotic activity, which corresponded to an inverse correlation between the two metrics (ρ = –0.05, *p* < 0.05). Conversely, in neutrophils, macrophages, and fibroblasts, higher pyroptotic activity was observed in the high score group and increased with rising PSAGS scores (Figure 7H,I). This cell type-specific inverse pattern suggests that high PSAGS score tumor cells may create a pro-pyroptotic immune microenvironment while resisting pyroptosis themselves. Further analysis within malignant ductal cells revealed that subgroup 1 had the highest PSAGS scores, followed by subgroups 2 and 3 (Figure S7E,F). Subgroup 2 exhibited significantly higher pyroptotic activity compared to subgroups 1 and 3 (Figure 7J). Functional enrichment analysis revealed that pathways involved in EMT, stemness, and proliferation showed high enrichment in the high PSAGS score subgroup 1 (Figure 7K). Subgroup 2 was associated with peptide transport and cell adhesion, while subgroup 3, characterized by low PSAGS scores, was primarily involved in inflammatory response and immune activation. These findings demonstrate that the PSAGS score captures intratumoral heterogeneity among malignant cells, providing cellular-level insights into the biological basis for this prognostic model.

### 3.8. Cell–Cell Communication Analysis Reveals an Immunosuppressive and Fibrotic Microenvironment Associated with PSAGS Score

**Figure 8:**
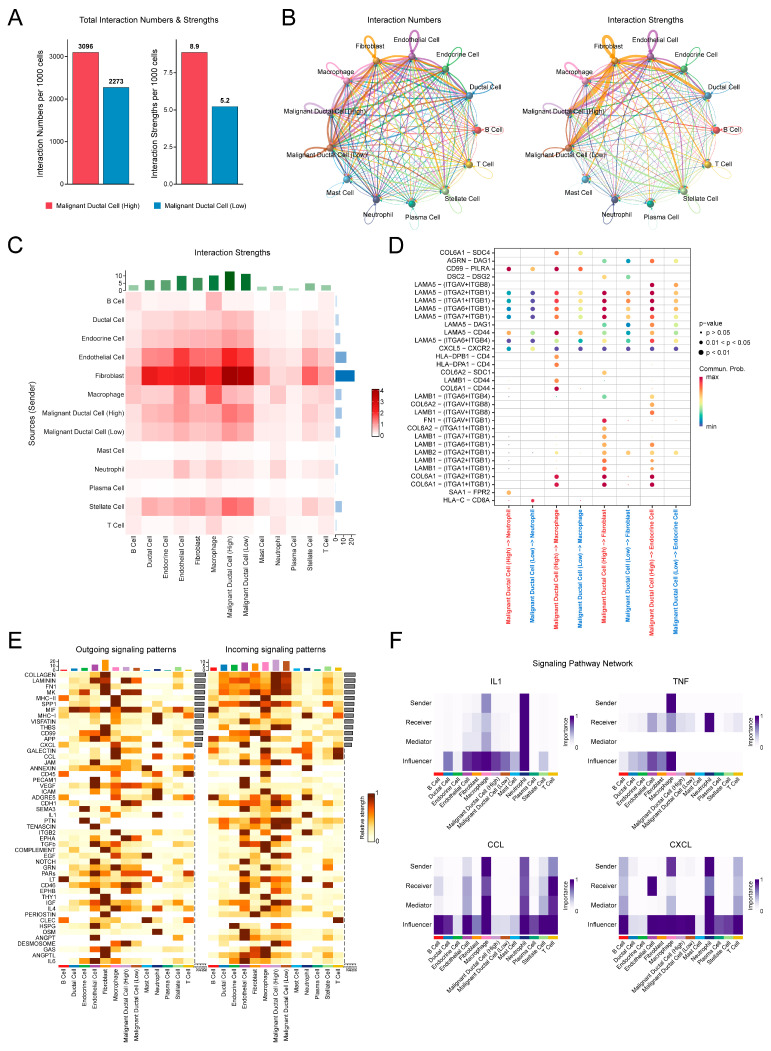
Cell communication analysis of microenvironmental characteristics associated with the PSAGS score. (**A**) Comparison of the number and strength of outgoing signals from high and low PSAGS malignant ductal cells to other cells. (**B**) Circle plot of interaction number and strength among all cell types in the microenvironment. (**C**) Heatmap of interaction strength among all cell types in the microenvironment. (**D**) Bubble plot of communication probability for differential ligand–receptor pairs across specific cell types, with the x-axis showing sender and receiver cells and the y-axis showing ligand–receptor pairs of signaling pathways. (**E**) Heatmap of outgoing and incoming signal flow for the top 50 pathways by signal strength in the microenvironment. (**F**) Signaling networks of IL1, TNF, CCL, and CXCL pathways.

We next sought to elucidate the potential mechanisms underlying PSAGS predictions by examining cell–cell communication between malignant ductal cells from the high and low PSAGS score groups and their surrounding microenvironment. When malignant ductal cells acted as signal senders, the group with a high PSAGS score exhibited more and stronger interactions than the group with a low score (Figure 8A). Shifting perspective to the entire network, we found that fibroblasts served as the dominant signal senders, while high PSAGS score malignant ductal cells were the primary signal receivers and received stronger signals than the low score group. Notably, both malignant ductal cell groups exhibited minimal communication with B cells, T cells, mast cells, or plasma cells (Figure 8B,C), indicating a common mechanism of immune evasion.

Given our prior observation that high PSAGS scores in certain cell groups correlated with elevated pyroptotic activity, we then focused on investigating the signaling initiated by malignant cell groups toward these specific populations. The high PSAGS score malignant cells showed stronger pro-fibrotic, adhesion-related, and inflammatory signaling with these cell populations (Figure 8D). Specifically, when signaling to macrophages, fibroblasts, and endocrine cells, pathways involving extracellular matrix (ECM) proteins such as COL6A1 and LAMB1, as well as integrin receptors like ITGAV and ITGB1, were enriched in groups with high PSAGS scores. In contrast, these signals were less common in those with low scores. In signals to neutrophils, the high-score group exhibited an elevated communication probability for inflammation-related ligand–receptor pairs, including SAA1-FPR2 and CD99-PILRA. In contrast, the low PSAGS score group demonstrated enhanced communication for the cytotoxic T cell activation-related pair, HLA-C-CD8A. Pathway-level analysis confirmed that fibroblasts and high PSAGS score malignant cells remained the strongest signal senders and receivers, respectively. ECM-related pathways such as COLLAGEN, LAMININ, and FN1 were strongly linked to malignant cells and exhibited stronger signal strength in the high PSAGS score group (Figure 8E). However, in networks of pyroptosis- and inflammation-related signaling pathways, including IL1, TNF, CCL, and CXCL, the dominant signal senders, receivers, and mediators were T cells, macrophages, and neutrophils, rather than malignant ductal cells (Figure 8F), underscoring the central role of immune cells in driving the inflammatory response within the TME. Thus, our findings indicate that high PSAGS score malignant cells may, through specific cell–cell interactions, actively shape a pro-fibrotic microenvironment lacking effective anti-tumor immune responses.

### 3.9. Multi-Omics Validation Reveals the Expression Patterns of PSAGS Genes

**Figure 9:**
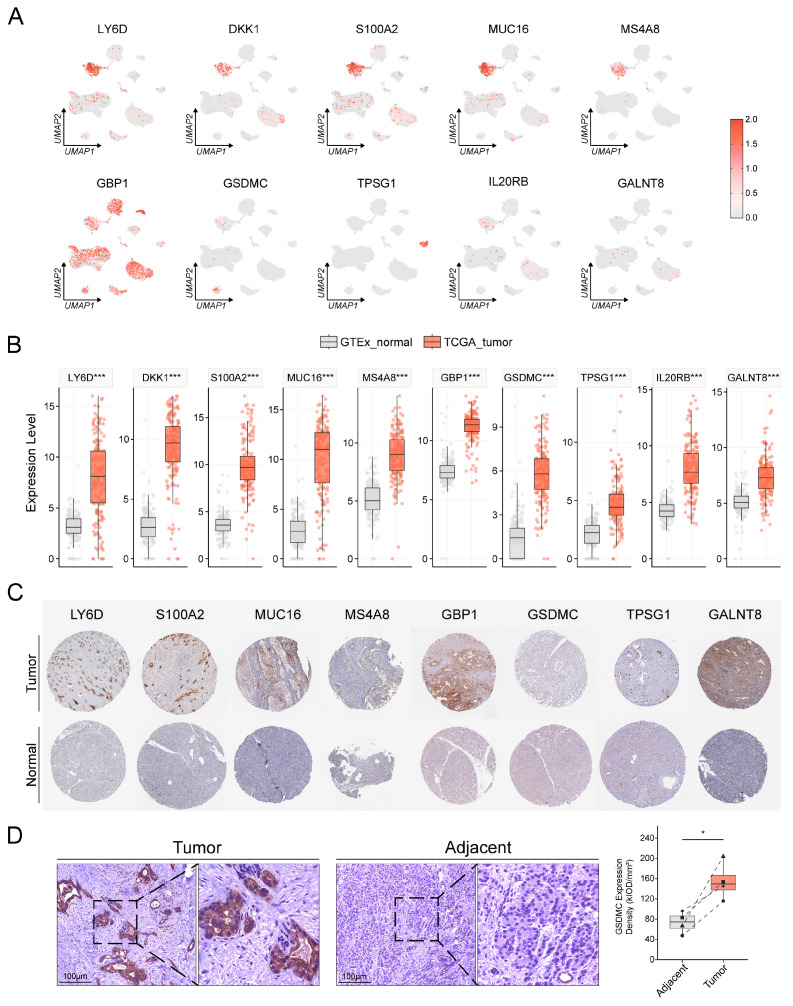
Multi-omics validation of PSAGS model gene expression patterns. (**A**) Model gene expression distribution in UMAP space from single-cell transcriptomics. (**B**) Model gene expression comparison between tumor and normal tissues from bulk transcriptomics. (**C**) Model gene protein expression comparison via the Human Protein Atlas (HPA) database immunohistochemistry images. Scale bar: 200 μm. (**D**) IHC images and quantitative analysis of GSDMC in pancreatic adenocarcinoma (PAAD) and adjacent normal tissues. Statistical methods: Wilcoxon rank-sum test (**B**); paired *t*-test (**D**); * (*p* < 0.05), *** (*p* < 0.001).

To confirm the biological basis of PSAGS, we performed multi-omics validation of its constituent gene expression. ScRNA-seq analysis revealed highly cell type-specific expression patterns: *LY6D*, *DKK1*, *S100A2*, *MUC16*, and *MS4A8* were enriched in ductal cells; *GSDMC* was co-expressed in ductal and endothelial cells; *TPSG1* was specifically localized to mast cells; and *GBP1* was broadly expressed across multiple cell types, whereas *IL20RB* and *GALNT8* were detectable but not significantly enriched in ductal cells (Figure 9A). Bulk RNA-seq analysis further corroborated a significant upregulation of all genes in PAAD tissues versus normal tissues (*p* < 0.001, Figure 9B). The HPA data (Figure 9C, Table S7) confirmed elevated protein expression levels in PAAD for seven genes: *LY6D*, *S100A2*, *MUC16*, *MS4A8*, *GBP1*, *TPSG1*, and *GALNT8*. For *DKK1*, *IL20RB*, and *GSDMC*, which yielded inconclusive results in the HPA due to lack of data or non-significant detection, high expression in PAAD has been independently reported in the literature [27,28,29]. We next performed immunohistochemical analysis of *GSDMC*, which demonstrated markedly increased expression in the tumor relative to matched adjacent normal tissues (154.8 ± 36.7 vs. 73.3 ± 21.1 kIOD/mm^2^, paired *t*-test *p* = 0.024, Figure 9D and Figure S8). Taken together, this multi-level evidence establishes a mechanistic foundation for the prognostic value of PSAGS.

## 4. Discussion

Globally, PAAD remains a major public health challenge, with rising incidence and limited therapeutic options [30]. Chemotherapy, targeted therapy, and immunotherapy can induce pyroptosis. This process stimulates anti-tumor immunity and alleviates immune suppression, offering a prospective approach to improve PAAD treatment [31]. For example, ponatinib and perifosin suppress PAAD progression by promoting pyroptosis through the caspase-3/GSDME pathway [32]. The combination of neobractatin and trametinib induces both apoptosis and pyroptosis via the ROS/AKT/GSDME axis, inhibiting PAAD growth [33]. It is also reported that chemotherapy-induced pyroptosis mediated by the same caspase-3/GSDME axis conversely enhances cancer cell invasion, migration, and chemotherapy resistance in PAAD [34]. These seemingly contradictory findings indicate that the TME, shaped by intratumoral heterogeneity [35] into one characterized by high immune and molecular diversity, likely constitutes the determinant background for the ultimate role of pyroptosis in PAAD. To address this, we employed multi-omics data and applied interpretable machine learning to develop and rigorously validate a prognostic model anchored in pyroptosis heterogeneity. Our study provides an alternative method for risk stratification and treatment decision-making previously undeveloped in PAAD, while offering high predictive accuracy and mechanistic transparency.

We integrated prognostic significance and differential expression to identify 16 key PRGs. These genes were then applied to stratify PAAD patients into two distinct subtypes. This stratification reflected the heterogeneity in pyroptosis between the clusters. Specifically, Cluster B showed evidence of higher basal pyroptotic activity relative to Cluster A, a phenotype marked by the notable upregulation of PRGs (e.g., *IL6*, *GSDMC*, *GBP1*, and *GZMA*). Despite showing stronger immune cell infiltration, Cluster B displayed features of a tumor-promoting microenvironment, including enrichment of the IL-6/JAK/STAT3, TNF-κB, and EMT pathways, a higher stromal score, and a relative deficiency of CD56dim NK cells, which collectively contribute to its poor prognosis. Mechanistically, inflammatory factors (e.g., *IL-18* and *IL-1β*) released during pyroptosis are key drivers of tumor inflammatory niche formation [36]. A chronic inflammatory microenvironment can drive malignant progression by activating pathways like IL-6/JAK/STAT, inducing EMT, and promoting cancer-associated fibroblasts (CAFs)-mediated immunosuppressive stromal remodeling [37]. Furthermore, the deficiency of CD56dim NK cells, the major cytotoxic NK subpopulation, is closely associated with an immunosuppressive microenvironment and poor prognosis [38]. Therefore, the poor prognosis of Cluster B can be attributed to a dysfunctional TME, where the pro-inflammatory potential of pyroptosis is co-opted into pro-tumor signaling, coupled with the loss of key effector immune cells. For such patients, future therapeutic strategies would likely require a multi-pronged approach combining targeted inhibition of inflammatory signaling, disruption of the stromal barrier, and immune reactivation.

Based on the systematic description of pyroptosis heterogeneity between subtypes, we developed a prognostic model named PSAGS, aiming to translate this biological finding into a precise clinical tool. Unlike traditional statistical modeling that relies on linear assumptions, this study employed the RSF algorithm to circumvent performance decline and interpretational bias due to model-mismatch effects when applied to tabular biomedical data, while also effectively capturing the intricate non-linear relationships and feature interactions inherent in high-dimensional genomic data [39]. This methodological advantage enabled PSAGS to perform excellently in internal validation and to demonstrate strong predictive performance across four test cohorts and five independent external validation cohorts. Its discriminative capacity across diverse cohorts and platforms validates the general significance of the biological patterns captured by the model, highlighting the core value of our methodology. Moreover, in comparison with 50 published models, PSAGS demonstrated superior performance, including more consistent cross-cohort validation and interpretable SHAP analysis, which are often lacking in previous models. This finding strongly confirms the feasibility and significant potential of translating the biological understanding of pyroptosis heterogeneity into a potent prognostic tool through advanced computational technologies.

Ensemble tree methods, including random forests and gradient boosted trees, are powerful predictive tools, but their black-box nature impedes deeper biomedical application [39]. Therefore, we employed SHAP to interpret the black-box mechanism of PSAGS, clearly revealing the hierarchical contribution of its 10 constituent genes to prognostic prediction. Specifically, *LY6D* was identified as the most influential risk-predictive feature and exhibited the highest mean absolute SHAP value. In PAAD, this molecule functions as a cancer stem cell-associated surface marker that enhances stemness, chemoresistance, and invasive metastasis [40]. Its high expression also marks a pre-existing drug-resistant cell subpopulation significantly associated with poor patient prognosis [41]. Although the predictive contributions of the PRGs *GSDMC* and *GBP1* did not rank among the top, they permeated the entire process from molecular subtype identification to prognostic model construction, serving as key bridges connecting the computational model to pyroptosis biology. In PDAC, *GSDMC* has been established as a key pro-oncogenic factor driving malignant progression through distinct mechanisms. On one hand, as a pyroptosis executioner protein, it promotes tumor proliferation and oxaliplatin resistance by activating the pentose phosphate pathway [29]. On the other hand, it can drive stemness and metastasis independently of pyroptosis through ADAM17-mediated cleavage and nuclear translocation [42]. *GBP1*, an important interferon-induced protein, drives invasion and metastasis by regulating the cytoskeleton, promoting immune evasion, and mediating chemoresistance in various malignancies, including glioblastoma and ovarian cancer [43]. The contributions of the remaining feature genes are also indispensable: *DKK1*, *IL20RB*, *S100A2*, and *MUC16* are risk factors associated with tumor proliferation, invasion, and immune regulation [27,28,44,45]. *GALNT8*, *MS4A8*, and *TPSG1* (a member of the same gene family as the mast cell markers *TPSAB1* and *TPSB2*) are suggested as potential protective factors whose expression levels correlate negatively with mortality risk [46,47,48]. Crucially, SHAP provided not only population-level mechanistic interpretation but also individual-level risk profiling. The heterogeneity of the core driver genes it revealed among patients provides a theoretical basis for future exploration of gene-tailored precision targeting strategies.

Multi-omics integration strategies have been established as a critical pathway to validate the biological basis of computational models and advance their clinical translation [49]. Following this paradigm, we performed multi-omics analyses to validate the biological rationality of the constituent genes of PSAGS. Namely, these 10 model genes showed significant tumor-specific upregulation in PAAD. Their distribution was highly cell type-specific. As shown in the single-cell transcriptomic atlas, risk factors (e.g., *LY6D*, *DKK1*, and *S100A2*) were specifically enriched in malignant ductal cells, consistent with their central predictive roles. Additionally, both authoritative HPA data [50] and our *GSDMC* immunohistochemistry results demonstrated consistent upregulation of these genes in tumor tissues. This convergent evidence confirmed that the molecular events underlying PSAGS are deeply rooted in the biology of PAAD. Thus, it provides a mechanistic explanation beyond pure statistics for the strong prognostic discriminatory ability of PSAGS.

The validated biological foundation of PSAGS supports its clear prospects for clinical application. Specifically, it can effectively identify patients likely to benefit from immunotherapy. Patients with low PSAGS scores are anticipated to respond better, likely attributable to a more immunoactive TME [51]. In terms of drug sensitivity, patients with high PSAGS scores exhibit heightened sensitivity to targeted agents, including dasatinib and trametinib. Preclinical studies confirm their mechanisms. Dasatinib reverses resistance to KRAS-G12C inhibitors and enhances anti-tumor efficacy by inhibiting SRC kinase and blocking JUN-mediated expression of the multidrug resistance protein ABCC1 [52]. Trametinib exerts its anti-cancer effects by suppressing the MAPK pathway, downregulating MYC, and activating the MiT/TFE–lysosome–iron metabolism axis [53]. On the other hand, patients with low PSAGS scores show greater sensitivity to chemotherapeutic drugs, including oxaliplatin and irinotecan. The underlying mechanism involves a profound synergy between the immunoactive microenvironment and chemotherapy-induced immunogenic cell death, which significantly augments anti-tumor immunity [54,55]. These findings provide a computational basis for personalized treatment aligned with pyroptosis heterogeneity: patients with high PSAGS scores could be prioritized for targeted therapy, whereas low-scoring patients might be recommended for oxaliplatin-containing chemotherapy. It should be noted, however, that these predictions are derived from computational models and are hypothesis-generating. Further prospective studies are needed to validate these predictions before any clinical application. Moreover, the nomogram model we developed by integrating the PSAGS score with independent prognostic factors demonstrates favorable calibration and clinical net benefit. This positions it as a promising candidate for direct translation into a clinically actionable and patient-specific decision aid.

Single-cell transcriptomic analysis provided unprecedented resolution to dissect the biology of PSAGS at the cellular level [56]. A major finding was the cell type-dependent inverse regulatory relationship between pyroptotic activity and PSAGS scores. Malignant ductal cells with high PSAGS scores exhibited lower pyroptotic activity, indicating that these cells may maintain a survival advantage by actively suppressing pyroptosis [57]. Meanwhile, neutrophils, macrophages, and fibroblasts in the TME showed high pyroptotic activity alongside elevated PSAGS scores. Notably, even though high PSAGS score malignant cells did not dominate classical pyroptosis-related inflammatory signaling (e.g., IL1 and TNF), they established enhanced communication networks with fibroblasts and macrophages via ECM protein (e.g., COL6A1, LAMB1)–integrin interactions. This activated ECM pathways (e.g., COLLAGEN and LAMININ) and fostered a pro-fibrotic stromal microenvironment [58]. Concurrently, the SAA1-FPR2 inflammatory signal from high PSAGS score malignant cells to neutrophils was also significantly amplified. This pathway is known to increase neutrophil-driven inflammation in inflammatory diseases [59]. These findings delineate a potential mechanistic framework wherein high PSAGS score malignant cells sustain survival by repressing intrinsic pyroptosis and, through active intercellular signaling reprogramming, induce both stromal fibrosis and immune cell pyroptotic inflammation in the microenvironment. This synergy ultimately generates an immunosuppressive microenvironment conducive to tumor progression, providing a compelling cellular-level rationale for the prognostic ability of PSAGS.

This study has several limitations. First, the development and validation of PSAGS, along with its therapeutic predictions, relied on retrospective data from public cohorts. These findings require confirmation in large prospective studies. Second, the precise biological roles of some model genes (e.g., *GBP1* and *TPSG1*) in PAAD remain unclear and need definitive characterization through gene editing and functional assays. Third, the insights from single-cell transcriptomic analysis are limited by the sample size, which makes them more exploratory in nature. Fourth, the immunohistochemical validation of *GSDMC* was based on only four paired PAAD samples, which limits the statistical power and generalizability of these findings. To strengthen these conclusions, expanding the sample size and incorporating experimental validation are necessary. Future work will focus on validating PSAGS in larger cohorts and elucidating the mechanisms of these genes through molecular and animal models, ultimately paving the way for the application of this model toward personalized therapy.

## 5. Conclusions

This study developed a prognostic model, PSAGS, by integrating interpretable machine learning with multi-omics validation. The model is grounded in pyroptosis heterogeneity and has established biological reliability. PSAGS demonstrated consistent prognostic ability across multiple cohorts, showing potential for clinical risk stratification and guiding personalized therapy in PAAD. Furthermore, we linked PSAGS scores to specific functional states of cells within the TME, providing a mechanistic explanation for the prognostic differences between patients. In summary, this work provides a novel analytical framework for PAAD prognosis and offers valuable insights into how pyroptosis heterogeneity influences tumor progression. Future research could leverage PSAGS to develop personalized treatment strategies that overcome the severe challenges in PAAD clinical management.

## Data Availability

The data presented in this study are available in publicly accessible repositories. These data were derived from the following resources available in the public domain: UCSC XENA database (https://xena.ucsc.edu/, accessed on 8 April 2026); TCGA GDC portal (https://portal.gdc.cancer.gov/, accessed on 8 April 2026); GEO database (https://www.ncbi.nlm.nih.gov/geo/, accessed on 8 April 2026); SangerBox platform (http://sangerbox.com/, accessed on 8 April 2026); ArrayExpress repository (https://www.ebi.ac.uk/arrayexpress/, accessed on 8 April 2026); cBioPortal database (https://www.cbioportal.org/, accessed on 8 April 2026); and MSigDB database (https://www.gsea-msigdb.org, accessed on 8 April 2026). Requests for further details should be addressed to the corresponding author.

## Supplementary Materials

The following supporting information can be downloaded at: https://www.mdpi.com/article/10.3390/biomedicines14040892/s1, Table S1. Dataset information used in this study. Table S2. Clinical characteristics of the TCGA-PAAD training cohort. Table S3. List of 90 PRGs. Table S4. List of 40 immune checkpoint genes. Table S5. Published prognostic signatures used for comparison. Table S6. Sixteen key PRGs for subtype construction. Table S7. Immunohistochemical validation of PSAGS genes from the HPA. Figure S1. Identification and functional analysis of DEPRGs. Figure S2. Kaplan–Meier survival curves of the 13 prognostic DEPRGs identified by univariate Cox regression analysis. Figure S3. Comparison of the distributions of survival status and clinicopathological characteristics between Cluster A and Cluster B. Figure S4. Gene selection workflow for the PSAGS model and its association with the molecular subtype. Figure S5. Distribution of patient survival time and status with increasing PSAGS score across all cohorts. Figure S6. Association of PSAGS with clinical characteristics and internal validation of the nomogram. Figure S7. Quality control of scRNA-seq data and characterization of malignant ductal cell subgroups. Figure S8. Representative IHC images of GSDMC expression in the remaining three paired PAAD tumor and adjacent normal tissues.

## Abbreviations

The following abbreviations are used in this manuscript:

BP	Biological Process
CAF(s)	Cancer-Associated Fibroblast(s)
CNV	Copy Number Variation
DCA	Decision Curve Analysis
DEG(s)	Differentially Expressed Gene(s)
DEPRG(s)	Differentially Expressed Pyroptosis-Related Gene(s)
DEPSG(s)	Differentially Expressed Pyroptosis Subtype-associated Genes
ECM	Extracellular Matrix
EMT	Epithelial–Mesenchymal Transition
FC	Fold Change
GO	Gene Ontology
GSVA	Gene Set Variation Analysis
HPA	Human Protein Atlas
IC50	Half-maximal inhibitory concentration
k	optimal cluster count
KEGG	Kyoto Encyclopedia of Genes and Genomes
KM	Kaplan–Meier
MAE	Mean Absolute Error
NMF	Non-negative Matrix Factorization
OS	Overall Survival
PAAD	Pancreatic Adenocarcinoma
PCA	Principal Component Analysis
PDAC	Pancreatic Ductal Adenocarcinoma
PPI	Protein–Protein Interaction
PRG(s)	Pyroptosis-Related Gene(s)
PSAGS	Pyroptosis Subtype-Associated Gene Signature
ROC	Receiver Operating Characteristic
RSF	Random Survival Forest
SHAP	SHapley Additive exPlanations
ssGSEA	single-sample Gene Set Enrichment Analysis
TIDE	Tumor Immune Dysfunction and Exclusion
TMB	Tumor Mutational Burden
TME	Tumor Microenvironment
t-SNE	t-distributed Stochastic Neighbor Embedding
UMAP	Uniform Manifold Approximation and Projection
