# Supplementary material for: From Pyroptosis Heterogeneity to an Interpretable Prognostic Signature for Risk Stratification and Therapy Insights in Pancreatic Adenocarcinoma
Source: Biomedicines. 2026 Apr 14;14(4):892. doi: 10.3390/biomedicines14040892 (PMC13113290; doi:10.3390/biomedicines14040892)
Supplement: Supplementary file 1 [file biomedicines-14-00892-s001.zip › Supplementary Material 1.pdf]

Supplementary Tables

Table S1. Dataset information used in this study.

Cohort	Sample size		Source	Country	Data type
	Tumor	Normal			
TCGA-PAAD	178	4	TCGA	America	Bulk transcriptome
TCGA TARGET GTEx	178	171	UCSC XENA	America	
ICGC-PACA-CA	264	0	SangerBox	Canada	
ICGC-PACA-AU	92	0	SangerBox	Australia	
GSE79668	51	0	NCBI-GEO	America	
GSE78229	50	0	NCBI-GEO	America	
GSE71729	114	0	NCBI-GEO	America	
GSE62452	64	0	NCBI-GEO	America	
GSE183795	129	0	NCBI-GEO	America	
E-MTAB-6134	309	0	ArrayExpress	Europe	
CPTAC-PAAD	140	76	cBioPortal	America	
GSE212966	6	6	NCBI-GEO	China	Single cell transcriptome

Table S2. Clinical characteristics of the TCGA-PAAD training cohort.

Characteristic	Variable	Number	Characteristic	Variable	Number
Survival Status	Alive	85	Sex	Female	80
	Dead	93		Male	98
Age	<=65	94	Tumor Size	<=4	123
	>65	84		>4	55
Stage	Stage I	21	Grade	G1	31
	Stage II	147		G2	95
	Stage III	3		G3	48
	Stage IV	4		G4	2
	NA	3		Gx	2
T	T1	7	Residual Tumor	R0	106
	T2	24		R1	53
	T3	142		R2	5

	T4	3		Rx	4
	Tx	1		NA	10
	NA	1	Tumor Location	Head	139
N	N0	49		Body	14
	N1	124		Tail	14
	Nx	4		Other	11
	NA	1			
M	M0	80			
	M1	4			
	Mx	94			

Table S3. List of 90 pyroptosis-related genes(PRGs).

AIM2	APIP	BAK1	BAX	CARD8	CASP1
CASP3	CASP4	CASP5	CASP6	CASP7	CASP8
CASP9	CFLAR	CHMP2A	CHMP2B	CHMP3	CHMP4A
CHMP4B	CHMP4C	CHMP6	CHMP7	CTSG	CYCS
DHX9	DPP8	DPP9	ELANE	FADD	FOXO3
GBP1	GBP5	GPX4	GSDMA	GSDMB	GSDMC
GSDMD	GSDME	GZMA	GZMB	HMGB1	IFNG
IL18	IL1A	IL1B	IL1R1	IL6	IRAK1
IRF1	IRF2	IRF6	IRF8	MAP3K20	MAP3K5
MAPK11	MAPK14	MAPK8	MEFV	NAIP	NEK7
NLRC4	NLRP1	NLRP2	NLRP3	NLRP6	NLRP7
NLRP9	NOD1	NOD2	NR2C2	P2RX7	PANX1
PJVK	PLCG1	PRKACA	PYCARD	RIPK1	RIPK3
SCAF11	STAT3	TGFB1	TIRAP	TLR4	TNF
TNFRSF1A	TP53	TP63	TRADD	TREM2	ZBP1

Table S4. List of 40 immune checkpoint genes.

ADORA2A	BTLA	BTNL2	CD200	CD200R1
CD244	CD27	CD274	CD276	CD28
CD40	CD40LG	CD44	CD48	CD70
CD80	CD86	CTLA4	HAVCR2	HHLA2
ICOS	ICOSLG	IDO1	LAG3	LAIR1

LGALS9	NRP1	PDCD1	PDCD1LG2	TIGIT
TNFRSF4	TNFRSF9	TNFRSF14	TNFRSF18	TNFRSF25
TNFSF4	TNFSF9	TNFSF14	TNFSF15	VTCN1

Table S5. Published prognostic signatures used for comparison.

Model	PMID	Gene Count	Model	PMID	Gene Count
Signature-Ba	39196457	5	Signature-Shi	38028544	10
Signature-Cai	33872662	2	Signature-Su	35677632	3
Signature-Chen[1]	38996145	4	Signature-Sun	38363947	3
Signature-Chen[2]	39208654	6	Signature-Tan	36269388	4
Signature-Chen[3]	34772375	7	Signature-Tao	36118860	5
Signature-Chen[4]	37024802	3	Signature-Wang[1]	37547756	5
Signature-Chen[5]	35938160	4	Signature-Wang[2]	38254861	4
Signature-Chen[6]	34090418	4	Signature-Wei	36685915	9
Signature-Chen[7]	31102348	4	Signature-Wu	39095628	4
Signature-Ding	35756487	3	Signature-Xie[1]	35682857	8
Signature-Du	37628967	6	Signature-Xie[2]	37782328	3
Signature-Feng	33996821	8	Signature-Xie[3]	35783277	4
Signature-Ge	36613599	5	Signature-Xu[1]	33176521	5
Signature-Hao	37333498	16	Signature-Xu[2]	36253973	5
Signature-Hu	39708151	4	Signature-Yan	33133266	4
Signature-Jiang	34816714	5	Signature-Yang[1]	36353587	4
Signature-Li	39430819	2	Signature-Yang[2]	35033072	3
Signature-Liu[1]	35035831	3	Signature-Yuan	34490033	3
Signature-Liu[2]	35195559	7	Signature-Zhang[1]	35785176	8
Signature-Liu[3]	34902987	2	Signature-Zhang[2]	37330494	12
Signature-Luo	35635957	5	Signature-Zhang[3]	33015155	5
Signature-Ma[1]	39332020	7	Signature-Zhang[4]	39604668	5
Signature-Ma[2]	34660806	6	Signature-Zhang[5]	39209222	5
Signature-Meng	32490170	16	Signature-Zhang[6]	35342420	4
Signature-Ren	37405638	6	Signature-Zou	34654352	4

Table S6. Sixteen key PRGs for subtype construction.

AIM2	BAK1	BAX	CASP3
CHMP4B	FADD	GBP1	GBP5
GPX4	GSDMC	IL18	IRF8
PANX1	PYCARD	TP63	ZBP1

Table S7. Immunohistochemical validation of model genes from the Human Protein Atlas.

Gene	ID	Pancreatic tissue	Staining	Antibody
LY6D	3764	tumor	high	HPA024755
	1647	normal	not detected	
S100A2	875	tumor	medium	CAB002600
	2220	normal	not detected	
MUC16	4400	tumor	medium	CAB055172
	4156	normal	not detected	
MS4A8	4555	tumor	medium	HPA007319
	2295	normal	not detected	
GBP1	4555	tumor	medium	CAB015450
	2562	normal	not detected	
GSDMC	4555	tumor	not detected	HPA026317
	1920	normal	not detected	
TPSG1	4924	tumor	medium	HPA060458
	1920	normal	not detected	
GALNT8	3851	tumor	high	HPA012638
	4156	normal	not detected	
IL20RB	No validation data			
DKK1				
