# Supplementary material for: From Pyroptosis Heterogeneity to an Interpretable Prognostic Signature for Risk Stratification and Therapy Insights in Pancreatic Adenocarcinoma
Source: Biomedicines. 2026 Apr 14;14(4):892. doi: 10.3390/biomedicines14040892 (PMC13113290; doi:10.3390/biomedicines14040892)
Supplement: Supplementary file 1 [file biomedicines-14-00892-s001.zip › Supplementary Material 2.pdf]

Supplementary Figures

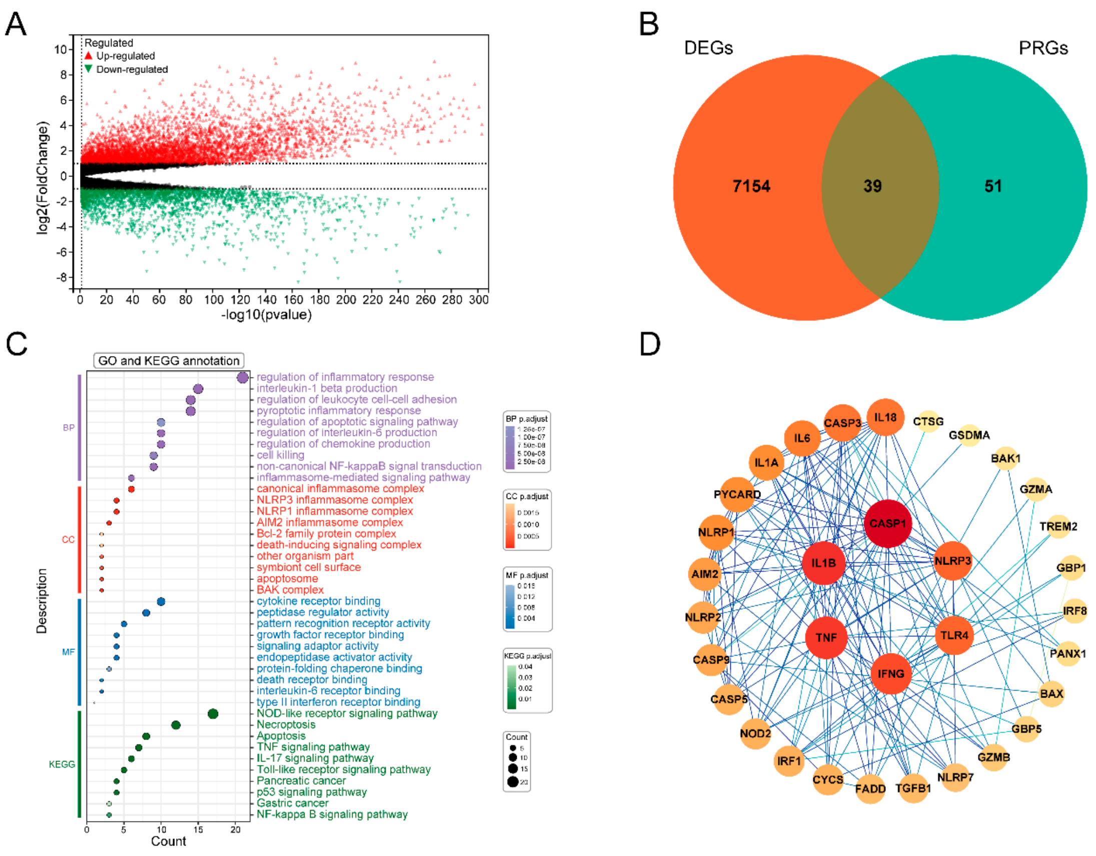

Figure S1. Identification and functional analysis of differentially expressed pyroptosis-related genes (DEPRGs).

(A) Volcano plot of differentially expressed genes (DEGs) between tumor and normal tissues. **(B)** Venn diagram of the intersection between DEGs and pyroptosis-related genes (PRGs). **(C)** Bubble plot of Gene Ontology (GO) terms and Kyoto Encyclopedia of Genes and Genomes (KEGG) pathways enrichment analysis for DEPRGs. **(D)** Protein-protein interaction network of DEPRGs.

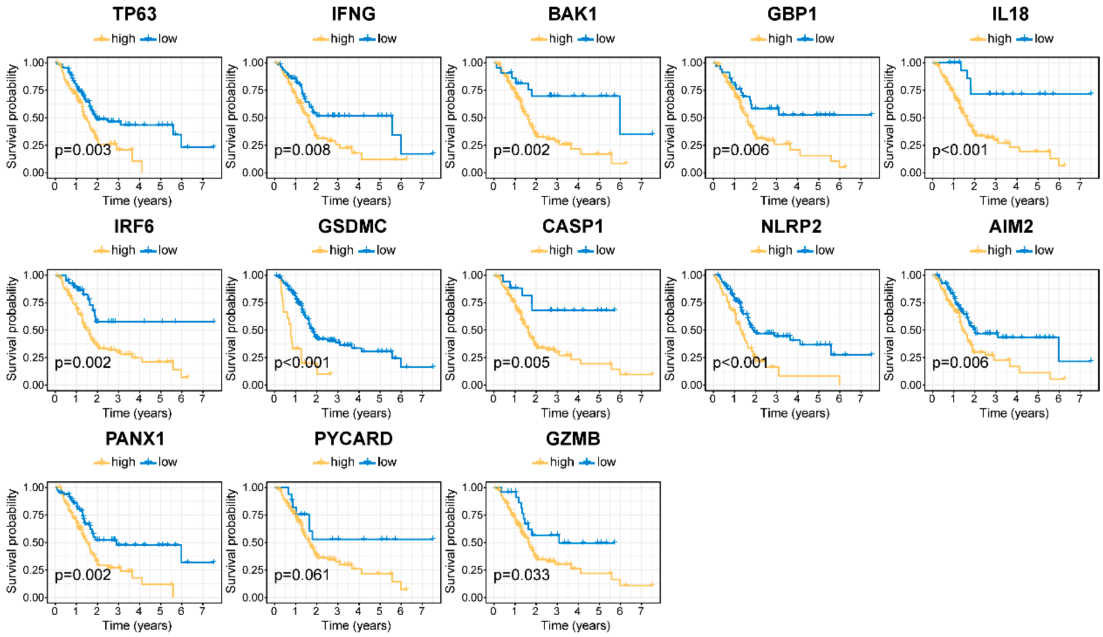

Figure S2. Kaplan-Meier survival curves of the 13 prognostic DEPRGs identified by univariate Cox regression analysis. Statistical methods: log-rank test.

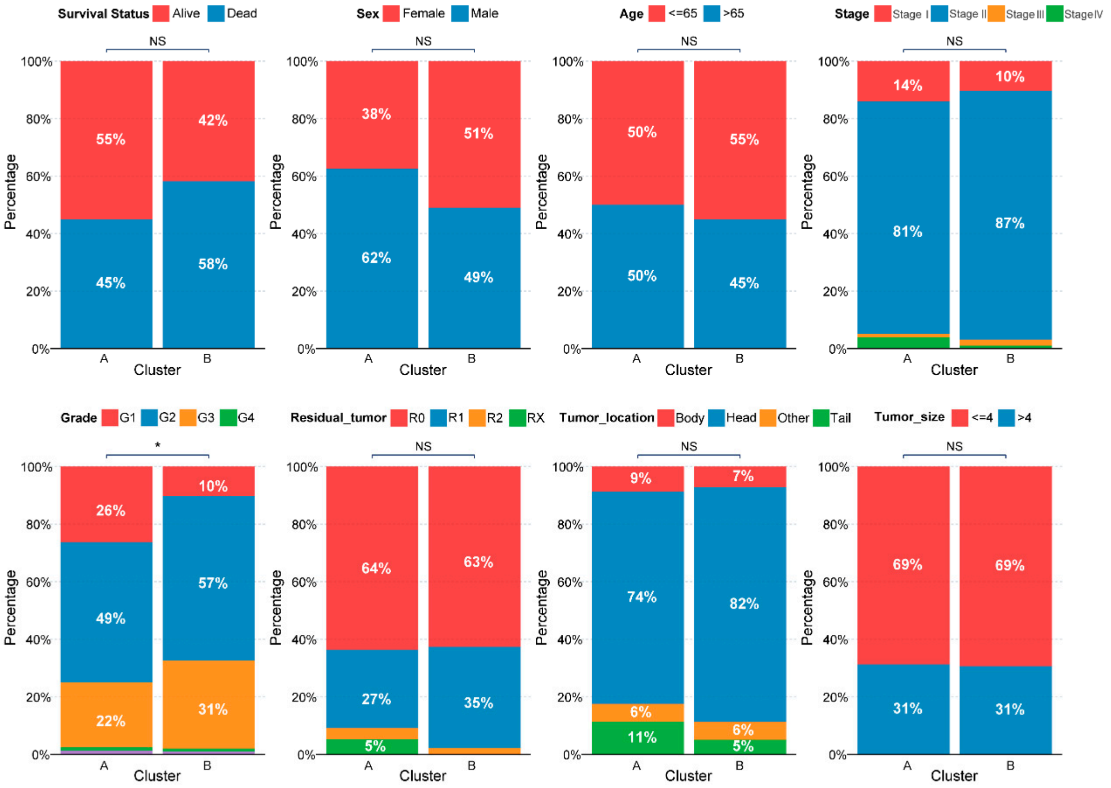

Figure S3. Comparison of the distributions of survival status and clinicopathological characteristics between Cluster A and Cluster B. Statistical methods: chi-square test (survival status, sex, age, tumor_size); Fisher's exact test (stage, grade, residual_tumor, tumor_location); ns ($p > 0.05$), * ($p < 0.05$), ** ($p < 0.01$), *** ($p < 0.001$).

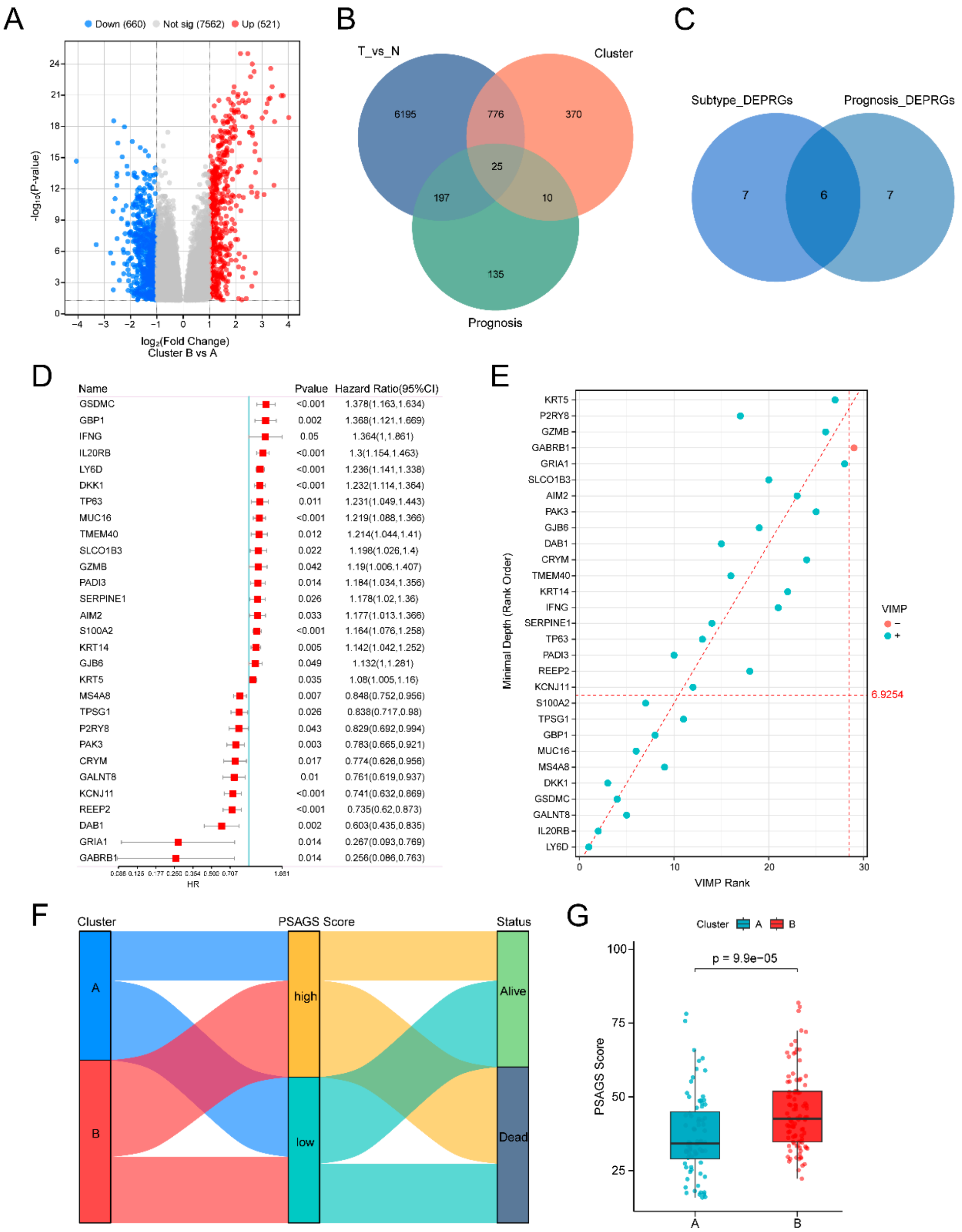

Figure S4. Gene selection workflow for the pyroptosis subtype-associated gene signature (PSAGS) model and its association with the molecular subtype.

(A) Identification of DEGs between Cluster A and Cluster B. **(B)** Venn diagram of the intersection among DEGs between tumor and normal tissues, differentially expressed pyroptosis subtype-associated genes (DEPSGs), and pancreatic adenocarcinoma (PAAD)-related prognostic genes. **(C)** Venn diagram of the intersection between prognostic DEPRGs and subtype-related DEPRGs. **(D)** Forest plot of the results from the univariate Cox regression analysis of candidate DEPSGs. **(E)** Schematic of model gene selection based on variable importance and minimal

depth. **(F)** Association among molecular subtypes, PSAGS scores, and survival status. **(G)** Comparison of PSAGS scores between clusters. Statistical methods: Wald test from univariate Cox regression (D); Wilcoxon rank-sum test (G).

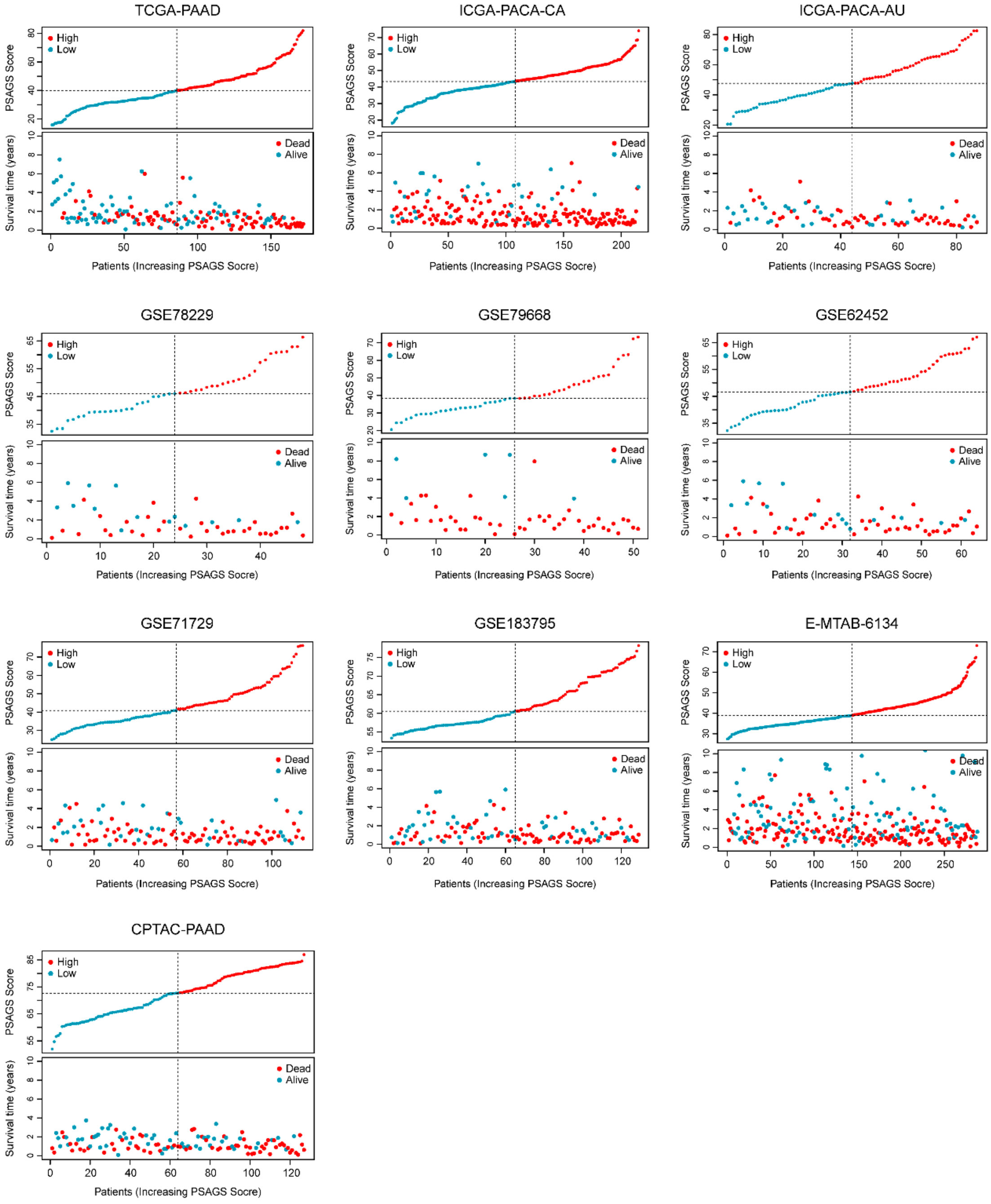

Figure S5. Distribution of patient survival time and status with increasing PSAGS score across all cohorts.

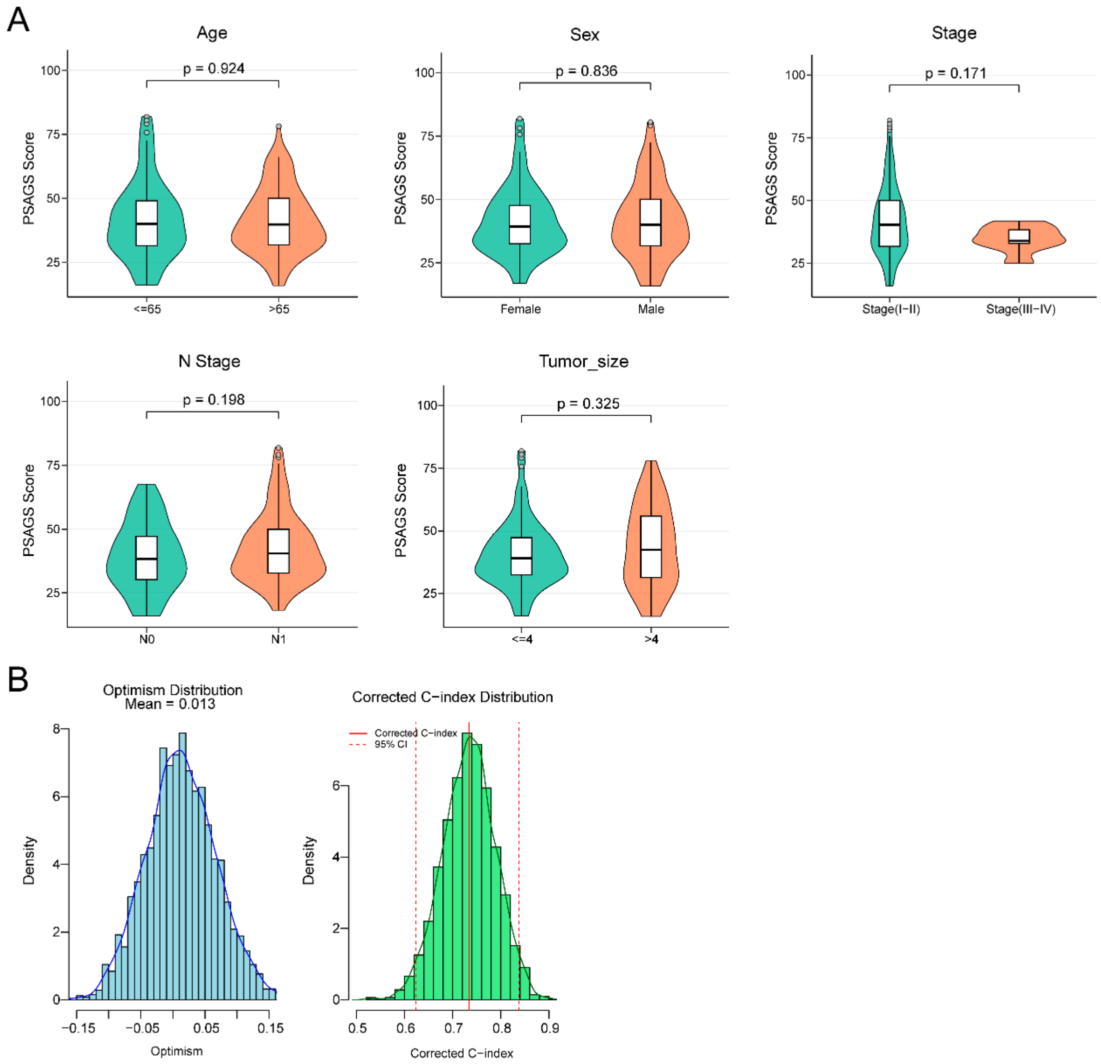

Figure S6. Association of PSAGS with clinical characteristics and internal validation of the nomogram.

(**A**) Comparison of PSAGS scores across groups stratified by age, gender, stage, N stage, and tumor size. (**B**) Distribution of the optimism and corrected C-index for the nomogram based on bootstrap validation in the training cohort. Statistical methods: Wilcoxon rank-sum test (A).

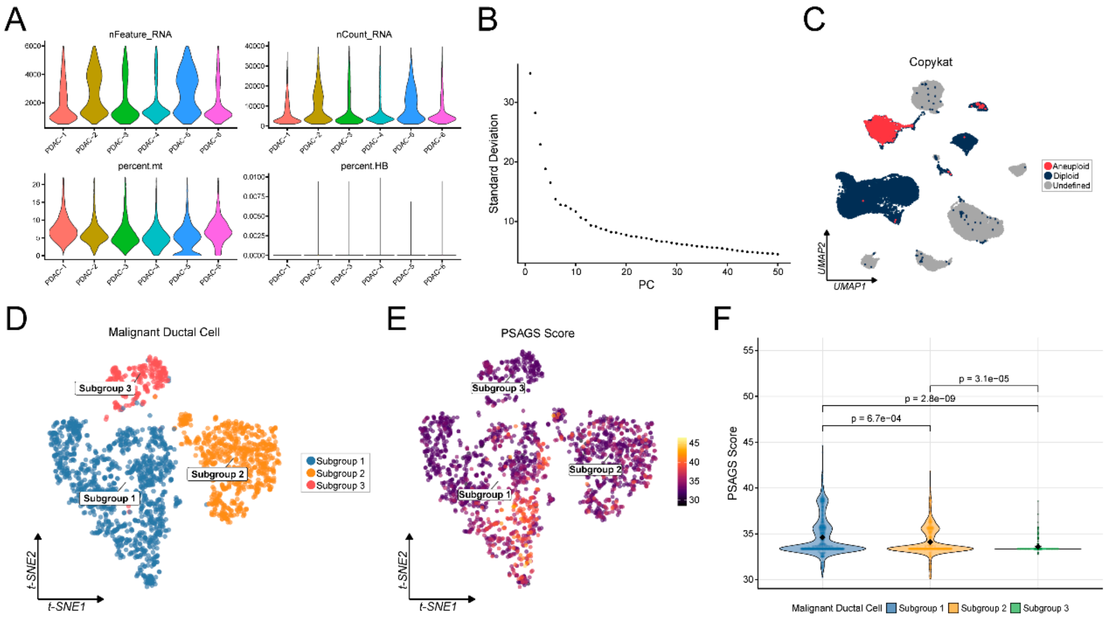

Figure S7. Quality control of single-cell RNA sequencing data and characterization of malignant ductal cell subgroups.

(A) Distribution of cells across quality control metrics after filtering. **(B)** Elbow plot for determining the dimensionality in principal component analysis. **(C)** Uniform manifold approximation and projection(UMAP) plot of malignant ductal cells inferred by the copykat method. **(D)** T-distributed stochastic neighbor embedding(t-SNE) plot of malignant ductal cell subgroups. **(E)** Distribution of PSAGS scores in t-SNE space across malignant ductal cell subgroups. **(F)** Comparison of PSAGS scores among malignant ductal cell subgroups. Statistical methods: Wilcoxon rank-sum test (F).

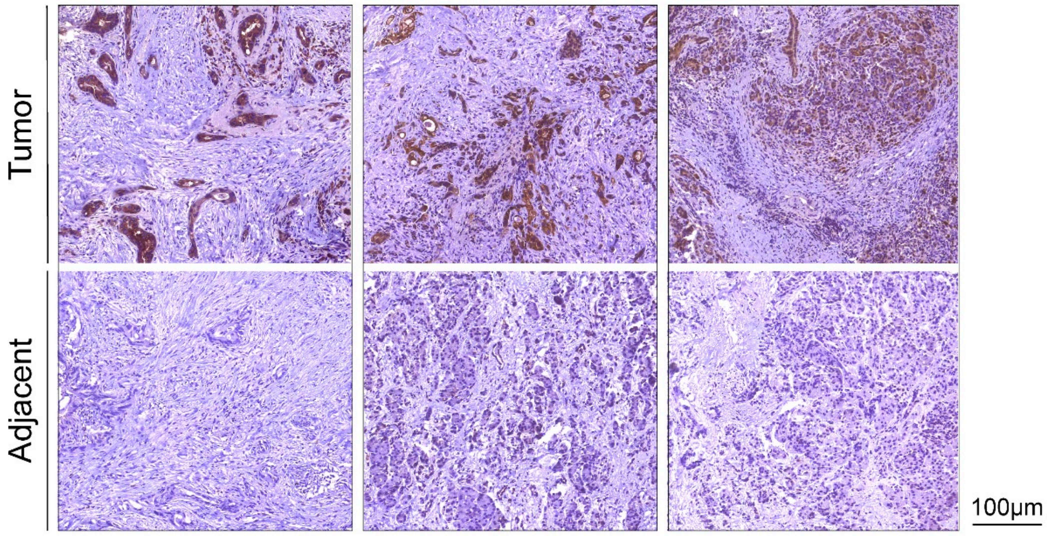

Figure S8. Representative IHC images of GSDMC expression in the remaining three paired PAAD tumor and adjacent normal tissues.
